# The influence of inflammation on the characteristics of adipose-derived mesenchymal stem cells (ADMSCs) and tissue repair capability in a hepatic injury mouse model

**DOI:** 10.1186/s13287-023-03532-z

**Published:** 2023-11-19

**Authors:** Jingfang Xiao, Xiaoyuan Gong, Zhenlan Fu, Xiongbo Song, Qinghua Ma, Jingya Miao, Ruili Cai, Zexuan Yan, Shuai Wang, Qian Li, Yaokai Chen, Liu Yang, Xiuwu Bian, Yemiao Chen

**Affiliations:** 1 Institute of Pathology and Southwest Cancer Center, Southwest Hospital, Army Medical University, Chongqing, People’s Republic of China; 2 Center for Joint Surgery, Southwest Hospital, Army Medical University, Chongqing, People’s Republic of China; 3 Biobank and Clinical Research Center, Chongqing Public Health Medical Center, Chongqing, People’s Republic of China

**Keywords:** Human infrapatellar fat pad, Mesenchymal stem cells, Inflammation, Characteristics

## Abstract

**Background:**

Mesenchymal stem cells (MSCs) are adult stem cells with self-renewal and multi-directional differentiation potential and possess the functions of immunomodulation, regulation of cell growth, and repair of damage. Over recent years, MSCs have been found to regulate the secretion of inflammatory factors and to exert regulatory effects on various lymphocytes in inflammatory states, and on the subsequent repair of tissue damage caused by inflammation. In the present study, we analyzed the effects of tissue inflammation on the characteristics of MSCs.

**Methods:**

Human fat derived from the infrapatellar fat pad (IPFP) of knees with differing degrees of inflammation was extracted from specimens derived from total knee arthroplasties. HE and immunohistochemical staining was performed to directly observe the evidence and degree of inflammation in human infrapatellar fat pad tissue in order to classify MSCs cells, by their origin, into highly inflamed and lowly inflamed groups, and to study the effect of tissue inflammation on cell acquisition rates via cellular counting data. Flow cytometry assays were performed to investigate the effect of tissue inflammation on MSC surface marker expression. Trilineage differentiation, including osteogenesis, adipogenesis, and chondrogenesis, was performed to assess the effect of tissue inflammation on the ability of MSCs to undergo directed differentiation. The effect of tissue inflammation on the ability of MSCs to proliferate was investigated via clone formation studies. RNA-sequencing was performed to evaluate the transcriptomes of MSCs derived from different areas of inflammation. The effect of tissue inflammation on tissue repair capacity and safety of MSCs was investigated via a murine model of acute liver injury.

**Results:**

The results of cell count data indicate that a high degree of tissue inflammation significantly decreases the acquisition rate of MSCs, and the proportion of CD34^+^ and CD146^+^ cells. The results of our trilineage differentiation assay show that a higher degree of inflammation decreases osteogenic differentiation and enhances adipogenic and chondrogenic differentiation of MSCs. However, these differences were not statistically significant. Clone formation assays indicate that the degree of tissue inflammation at the MSC source does not significantly affect the proliferative capacity of MSCs. The transcriptomes of MSCs remain relatively stable in fat pad tissues derived from both highly and lowly inflamed samples. The results of acute liver injury investigations in mice indicate that MSCs of high and low inflammatory tissue origin have no significant difference in their tissue repair capability.

**Conclusions:**

High tissue inflammation at the source of MSCs reduces the acquisition rate of MSCs and the percentage of CD34^+^ and CD146^+^ cells acquisition. However, source tissue inflammation may not significantly affect trilineage differentiation potential and proliferative capacity of MSCs. Also, MSCs obtained from differing source degrees of inflammation retain stable and similar transcriptomic profile and are both safe and efficacious for tissue repair/regeneration without detectable differences.

**Graphical Abstract:**

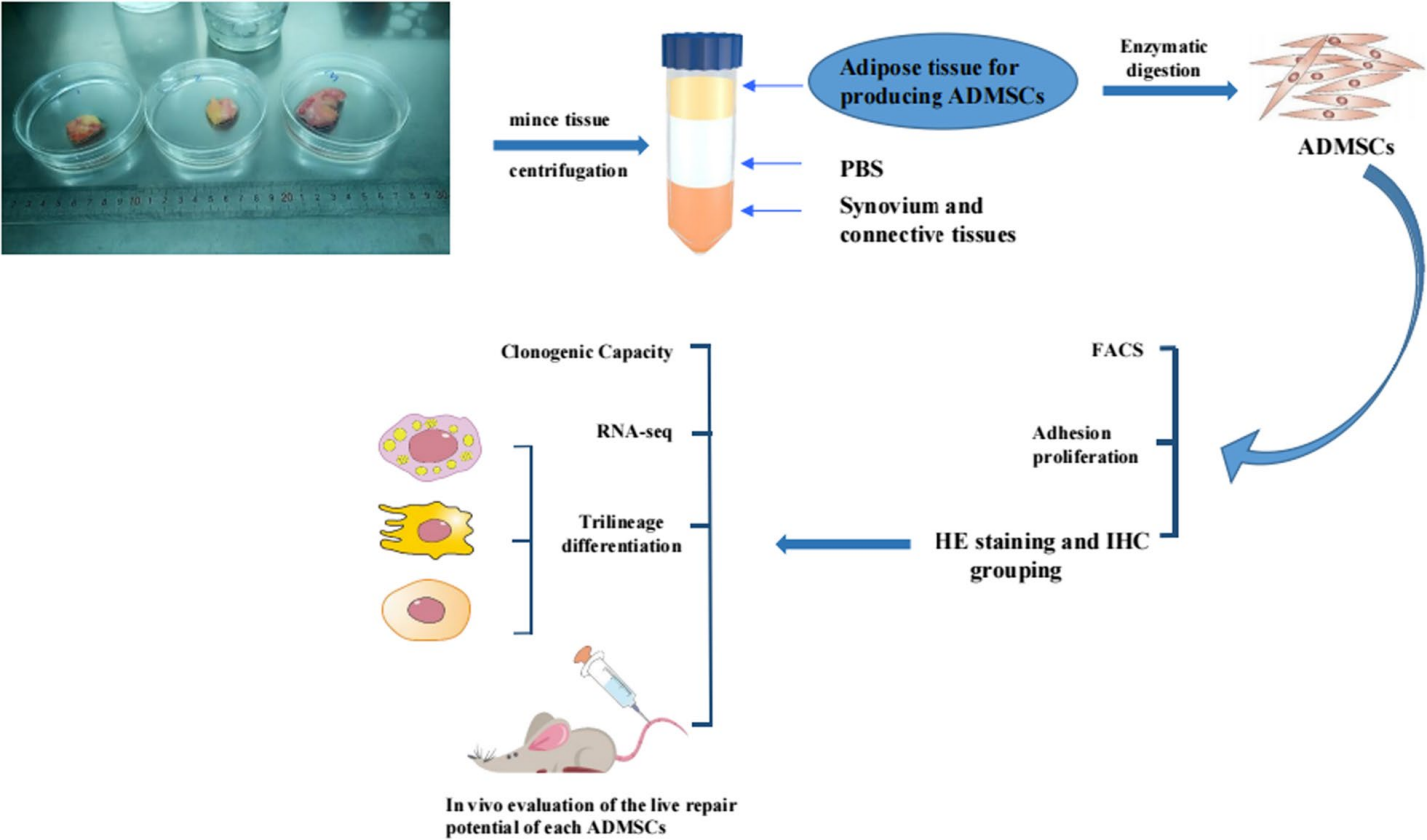

**Supplementary Information:**

The online version contains supplementary material available at 10.1186/s13287-023-03532-z.

## Background

Mesenchymal stem cells (MSCs) are derived from mesenchymal stroma, are adult stem cells with enhanced proliferative capacity, and these cells possess multi-directional differentiation potential. MSCs have been widely used for tissue regeneration as an engineered stem cell source with the potential to differentiate into osteoblasts, chondrocytes, adipocytes, bone marrow stroma, liver cells, islet cells, myocardial cells, and phenotypically neuron-like cells [1–5]. MSCs also possess immunomodulatory capabilities and may exert immunomodulatory functions through the secretion of soluble factors [such as interleukin 1, interferon gamma, tumor necrosis factor alpha, indoleamine 2, 3-dioxygenase, prostaglandin E2, inducible nitric oxide synthase, transforming growth factor-β1 (TGF-β1), hepatocyte growth factor, vascular endothelial growth factor, platelet-derived growth factor, insulin-like growth factor 1, stromal cell-derived factor 1, angiopoietin 1, interleukin 10, interleukin 6, and nitric oxide], or induce repair of tissue damage through cell to cell contact [6, 7].

Adipose tissue originates from the embryonic mesodermal germ layer; however, recent animal studies have observed that adipose tissue present at different anatomical locations may be of differing embryonic origins, and that individual fat deposits may not be functionally identical [8]. The presence of MSCs in adipose tissue was first reported by Zuck et al., in 2001. Compared to umbilical cord stem cells, adipose tissue-derived MSCs have been shown to have a greater differentiation capacity in preclinical studies [9–11]. Adipose-derived mesenchymal stem cells (ADMSCs) have been considered as the most promising candidates for stem cell therapy due to their wide accessibility and high regenerative potential [12–14].

The infrapatellar fat pad (IPFP) is a rich source of MSCs [9]. The presence of IPFP-MSCs in the knee joint has been recently reported and is now being actively studied [15–19]. The IPFP, a yellowish intracapsular fatty structure within the knee joint, is a mass of fibrous adipose tissue located within the capsule and outside the synovial membrane of the knee joint. It lies between the patellar tendon, the femoral condyle, and the tibial plateau of the knee joint, consists mainly of synovial membrane and subsynovial adipose tissue, and is an expansion of the structure and function of the synovial area of the knee [4, 20–22]. The IPFP belongs to the subcutaneous adipose tissue, is viewed as an endocrine and metabolic reservoir, and contains fewer inflammatory cells compared to visceral adipose tissue [23, 24].

Specific markers are ideal for the accurate identification of specific cells; however, surface markers associated with MSCs have not, as yet, been fully defined. To date, many surface markers have been proposed for MSCs, and the accepted view is that MSCs express CD105, CD73, CD44, CD90, CD71, Stro-1, and CD146, as well as the adhesion molecules CD106, CD166, ICAM-1, and CD29; however, MSCs do not express hematopoietic cell surface markers, such as CD45, CD34, and CD14, and do not express CD80, CD86, CD40, CD31, CD18, CD19, CD11b, and CD56 [9, 25–27].

There is growing evidence that MSC/progenitor cell-derived stem cells extracted from the infrapatellar fat pad exhibit unique specificity in terms of proliferative capacity, multilineage differentiation potential, and availability [4, 5, 28]. The ability of adult stem cells to proliferate and directionally differentiate is the basis for therapeutics related to tissue and organ regeneration and repair. An increasing number of studies have taken advantage of MSCs to conduct cell-based clinical trials on a variety of diseases, including liver diseases. Yan et al. have successfully isolated MSCs from human umbilical cords and have demonstrated that the use of human umbilical cord MSCs (huMSCs) may ameliorate liver injury and acute renal failure in mice [29–34].

Due to its unique anatomical location, the IPFP is sometimes involved in pathological states such as Hoffa’s disease, post-arthroscopy fibrosis, and limited nodular synovitis [35]. It has been shown that inflammation may influence the proliferation and differentiation of adult stem cells, such as inhibition of the multidirectional differentiation of bone marrow MSCs and periodontal stem cells, and promotion of the proliferative potential of adipose MSCs and neuron-like cells [36]. Thus, inflammation seems to have an appreciable influence on the ability of adult stem cells to effect tissue regeneration and repair, and the influence of inflammation on the proliferation and differentiation of adult stem cells leads to variations in the ability of adult stem cells to effectively repair tissue and competently achieve complete tissue regeneration.

The fate of stem cells may depend on their location of origin [37]. Some studies have shown that the biological properties of inflammatory tissue-derived stem cells are similar to those of healthy tissue-derived stem cells, while other investigations have observed that some of the properties of inflammatory tissue-derived stem cells are significantly impaired relative to healthy tissue-derived stem cells, especially their osteogenic differentiation and immunomodulatory capacity, and are detrimental to their tissue regenerative applications [38–40]. IPFPs having varying degrees of inflammation are commonly excised as surgical waste during knee arthroplasty, and IPFP tissue removed during surgery has considerable regenerative capacity (similar to synovium), has a lower degree of invasiveness, and induces fewer complications than bone marrow-derived MSCs. At the same time, the clinical translation of IPFP-derived MSCs (IPFSCs) is largely facilitated by the fact that the IPFP is derived from clinically discarded inflamed tissue, and thus faces relatively few ethical controversies, and is easily acceptable to patients. Therefore, in regenerative medicine, it is eminently feasible to obtain large amounts of IPFP tissue without significant adverse effects [2, 22, 41]. However, whether the degree of inflammation inherent in the IPFP tissue materially affects the proliferation, differentiation, and tissue repair capacity of its source MSCs has not, as yet, been studied. Therefore, in this investigation, surgical specimens from arthroscopic knee procedures were chosen as the source of inflamed adipose tissue from which to extract MSCs, to study the effect of tissue inflammation on the biological behavior of MSCs, thus providing a theoretical background for the study of autologous tissue repair via adipose-derived mesenchymal stem cells (ADMSCs).

## Methods

### Donor selection

Samples were obtained intraoperatively from total knee arthroplasties in 96 patients enrolled at the First Affiliated Hospital of Army Medical University. Written informed consent was obtained from the patients or their guardians. This study was performed following the principles of the Helsinki Declaration and was approved by the Ethics Committee of the First Affiliated Hospital of Army Medical University (KY2020044).

### Animal maintenance and treatment

The study mice were purchased from the Center of Animals (Army Medical University, Chongqing, China). Animal experimentation for our investigation was approved by the Institutional Animal Care and Use Committee of Army Medical University, in accordance with guidelines provided in the Guide for the Care and Use of Laboratory Animals. All experiments were designed and reported in accordance with the Animal Research: Reporting of In Vivo Experiments (ARRIVE) guidelines. Age-matched littermate mice were used in all the experiments. Six-week-old female C57 BL/6 mice were used as the study mice.

### Cell isolation

This study was performed at the Pathology and Southwest Cancer Center, the First Affiliated Hospital, Army Medical University, Chongqing, China. Samples were obtained intraoperatively from total knee arthroplasties in 96 patients (Male: 26, Female: 70, Age: 15–82), after receiving approval from the hospital ethics committee and obtaining informed consent from participants. For the isolation of ADMSCs, infrapatellar fat pad specimens (1.2–20.3 g) were obtained and washed three times in phosphate-buffered saline (Origene) supplemented with 1% penicillin/streptomycin (P/S; Beyotime), and then cut into pieces in cold PBS. After centrifugation, three layers were apparent, i.e., layer 1, settled at the bottom was synovium and connective tissue, layer 2 was PBS, and the topmost layer 3 was adipose tissue containing MSCs. Layer 3 was then immediately transferred to a new centrifuge tube (FALCON) and digested in digest medium (type II collagenase: 50 mg/mL tissue, Sigma) for 50 min at 37 ℃ with continuous shaking, and termination of digestion was performed in Dulbecco’s modified Eagle’s medium (DMEM, Gibco), supplemented with 10% fetal bovine serum (FBS; Lonza). After centrifugation at 600*g* for 10 min, the supernatant was discarded and the cell pellets were combined in 20 mL DMEM (+ 10% FBS). The resulting cell suspension was filtered through a 100 μM cell strainer (BD Bioscience). The collected cells were centrifuged at 600*g* for 5 min and resuspended in Red Cell Lysis Buffer (Beyotime) at room temperature for 5 min. The cells were then centrifuged again and resuspended in DMEM/F-12 (Invitrogen) supplemented with 20% FBS and 1% P/S. Cells were then filtered through a 40 μm cell strainer, and the viability of cells was ascertained by staining with Trypan Blue (Beyotime).

### Cell culture

Freshly isolated cells were washed twice with PBS and resuspended in FACs media (FBS with 2% FcR Blocking Reagent, human, Meltenyi Biotec). Pericyte and adventitial cells were sorted out by incubation with fluorochrome-conjugated mouse anti-human antibodies, i.e., FITC Mouse Anti-Human CD146 (BD Biosciences, 1:100), PE Mouse Anti-Human CD31 (BD Biosciences, 2:100), PE Mouse Anti-Human CD45 (BD Biosciences, 2:100), APC Mouse Anti-Human CD34 (BD Biosciences, 2:100), with immunoglobulin IgG1 isotype controls (BD Biosciences, 2:100) at 4 ℃ for 20 min in the absence of light, and running FACs following published protocols[42–44]. After washing in PBS, cells were screened via flow cytometry (Beckman Coulter Moflo XDP) and analyzed by Submit 5.2 software. IPFSCs were cultured in DMEM/F-12 supplemented with 1% PS and 20% FBS and incubated at 38 °C in a 5% CO_2_ humidified environment. Nonattached cells were washed off with PBS and adherent cells were maintained in dishes after 24 h. For all of the following experiments, IPFSCs from passages 4–6 were used in this study.

### Flow cytometry for MSC surface marker expression

All samples were studied for the expression of MSC surface markers by flow cytometry and a Human MSC Analysis Kit (BD Biosciences). IPFSCs were identified by the positive expression of CD105, CD73, CD44, and CD90, and the negative expression of CD34, CD11b, CD19, CD45, and HLA-DR. Passage 6 cells were washed in PBS and digested by trypsin. The harvested cells were resuspended at a concentration of 1 × 10^6^ cells/mL in FACS media and then incubated individually with FITE Mouse Anti-Human CD90, PE Mouse Anti-Human CD44, PerCP-Cy^TM^5.5 Mouse Anti-Human CD105, APC Mouse Anti-Human CD73, hMSC Positive Isotype Control Cocktail, hMSC Negative Isotype Control Cocktail, hMSC Positive Cocktail, hMSC Negative Cocktail, and nothing (100 μL per tube) at 4℃ for 30 min in the absence of light. The incubated cells were washed twice with PBS and resuspended at 300 µL in PBS. Cells were then analyzed on a flow cytometer (BD FACS Calibur). Data were analyzed using FlowJo software (Treestar).

### HE staining and IHC

Fresh tissues were embedded in OCT compound (Leica-microsystems) and frozen. The specimen was sectioned into 8 µm-thick sections and stained with hematoxylin and eosin (HE). For immunohistochemistry, the specimen was fixed for 20 min in cold acetone. After washing with PBS three times and blocking with goat serum (BOSTER) for 30 min, the specimen was individually incubated overnight with CD3, CD20, and CD68 (ZSGB-BIO) at 4 ℃ in the absence of light. Specimens were washed three times with PBS and incubated with secondary immunofluorescent antibodies, i.e., Rabbit/Mouse (DAKO). Nuclei were stained with 4’,6-diamidino-2-phenylindole (1:1000, Sigma-Aldrich) for one hour. Slides were then washed with PBS three times and sealed with neutral resin. Images were viewed, taken, and stored using an Olympus microscope. For CD68 macrophage counting, five representative areas (magnification × 400) were randomly selected on each slide and photographed with a BX51 microscope (Olympus). The area of each region and the corresponding number of CD68^+^ macrophages were measured using Image-Pro Plus 5.0 software (Media Cybernetics). Data are expressed as cells/mm^2^.

### Cell clone formation

MSCs were plated at 1000 cells/10 cm^2^ dishes (In Vitro Scientific). During colony growth, the culture medium was replaced every 3 d. After 14 days of culture, the cells were rinsed with PBS, fixed with 4% paraformaldehyde (Solarbio) for 20 min, and then stained with 0.1% crystal violet (Solarbio) for 20 min. Colonies containing more than 50 cells were counted and data collated.

### Trilineage differentiation of MSCs

MSCs were trypsinized and harvested from passage 6. For adipogenic differentiation, MSCs were plated into 24-well plates at 30,000 cells/well and cultured in adipogenic induction medium (Lonza) at 37 °C and 5% CO_2_. The medium was replaced every 2 days. After 21 days, cells were fixed in 4% paraformaldehyde for 30 min and stained by Oil Red O solution for 30 min. The cells were washed three times with PBS and viewed on an Olympus microscope. For osteogenic differentiation, MSCs were plated into 24-well plates at 30,000 cells/well and cultured in osteogenic induction medium (Lonza) at 37 °C and 5% CO_2_. The medium was replaced every two days. After 21 days, the cells were fixed with 4% paraformaldehyde for 30 min and stained with Alizarin Red S for 30 min. The cells were washed with PBS three times and viewed on an Olympus microscope. For chondrogenic differentiation, 1 × 10^6^ MSCs were resuspended in DMEM/F-12 medium with 20% FBS and centrifuged at 800 rpm for 5 min in a conical tube. The sediment was cultured at 37 °C and 5% CO_2_. After 24 h the supernatant was removed and 1 mL of chondrogenic induction medium was added to the tube. The bottom of the tube was gently flicked to suspend the cell ball in the medium and this system was maintained in an incubator. The medium was replaced every 2 days. After 21 days, the cell ball was fixed with 4% paraformaldehyde for 12 h and then embedded in paraffin. The specimen was shaved into 8 µm-thick sections and stained with Alcian blue. Images were then viewed, taken, and stored using an Olympus microscope.

### mRNA isolation and RNA-sequencing

MSCs were cultured at 1 × 10^5^ cells per well in DMEM/F-12 supplemented with 20% FBS and 1% P/S, and the medium was changed every 2 days. After 5 days, the culture medium was removed and the cells were rinsed with PBS. The total RNA was isolated with TRizol reagent (Life Technologies). RNA-sequencing of the MSCs was performed by BGI, China (http://www.genomics.cn/). Sangerbox (http://sangerbox.com/index.html) and Dr.Tom (BGI.Tech) software was utilized to analyze and describe the gene expression characteristics of the MSCs.

### In vivo regeneration of CCl_4_-induced liver injury

The C57 BL/6 mice received intraperitoneal injections of a 10% solution of CCl_4_ (10 µL/g) dissolved in corn oil once per day on two consecutive days. The mice were subsequently injected with 1 × 10^6^ MSCs in 100 µL PBS or 100 µL of PBS (control) via the tail vein. The study mice were euthanized two weeks after MSC transplantation by cervical cord dislocation and the livers were harvested and snap frozen in liquid nitrogen, and stored at − 80 °C. The tissues were embedded in OCT compound and frozen. The specimens were shaved into 6 µm sections and stained with hematoxylin and eosin (HE) for assessment of in vivo toxicity and the regenerative capability of MSCs.

### RNA isolation and qRT-PCR

Total RNA was extracted and purified from cells and tissues using TRizol reagent (Life Technologies). cDNA was synthesized with PrimeScript™ RT Master Mix (TakaRa). Quantitative real-time PCR (qRT-PCR) was used to analyze the gene expression of Trilineage Differentiation markers. For analysis of the gene expression of adipogenic markers, primers of CEBPA (CCAAT/enhancer binding protein, alpha), FABP4 (Fatty acid binding protein 4), and PPAGR2 (Peroxisome proliferator-activated receptor gamma 2) were used. For osteogenic markers, primers of ALPL (Alkaline phosphatase from liver/bone/kidney), Ocn (osteocalcin), and Runx2 (Runt-related transcription factor 2) were used. For chondrogenic markers, primers of ACAN (Aggrecan) and Sox 9 (Sex-determining region Y-box 9) were used. GADPH was used as an endogenous control. For determination of therapeutic effect for the liver injury, primers of GST (Gamma glutamyl transpeptidase), ALB (Albumin), Nrf-2, Hmox-1, and CK19 were used. qRT-PCR was performed using the SYBR Green PCR Master Mix (TakaRa). Primer sets are shown in Table 1.

**Table 1:** Primers used in the study

Genes	Forward (5′–3′)	Reverse (5′–3′)
CEBPA	CCTTGTGCCTTGGAAATGCAAAC	CTGCTCCCCTCCTTCTCTCA
FABP4	TCAGTGTGAATGGGGATGTGAT	TCTGCACATGTACCAGGACACC
PPAGR2	GCGATTCCTTCACTGATACACTG	GAGTGGGAGTGGTCTTCCATTAC
ALPL	CCAACGTGGCTAAGAATGTCATC	TGGGCATTGGTGTTGTACGTC
Ocn	CACTCCTCGCCCTATTGGC	CCCTCCTGCTTGGACACAAAG
Runx2	TGGTTAATCTCCGCAGGTCAC	ACTGTGCTGAAGAGGCTGTTTG
ACAN	AGGAGCAGGAGTTTGTCAACAAC	AGTTCTCAAATTGCATGGGGTGT
Sox 9	CTGGGCAAGCTCTGGAGA	ATGTGCGTCTGCTCCGTG
Human GADPH	TGCACCACCAACTGCTTAGC	GGCATGGACTGTGGTCATGAG
GST	GGCAGCCAAACCTAAGCTC	CCCTGGTCTGTGTCAGCATC
ALB	TCGCTACACCCAGAAAGCAC	CAGCAGACACACACGGTTCAG
Nrf-2	TGGGTTCAGTGACTCGGAAA	GACCAGGACTCACGGGAACT
Hmox-1	TACCTTCCCGAACATCGACA	TCTGCAGGGGCAGTATCTTG
CK19	TGCTGAAGCCACCTACCTTG	ATACTCCTGGTTCTGGCGCT
Mouse GADPH	GTCTTCTCCACCATGGAGAAGGCT	CATGCCAGTGAGCTTCCCGTTCA

### Statistical analyses

All data were analyzed by PASW Statistics for Windows, Version 18 (SPSS Inc. IBM Corp., Chicago, IL, USA) or GraphPad Prism (version 8.3.0; San Diego, CA, USA). All quantified data were statistically analyzed. Parametric tests were used for normally distributed data, while non-parametric tests were used to analyze data of small samples (such as trilineage differentiation and clone formation assay). A two-tailed *p* value of less than 0.05 was considered statistically significant.

## Results

### Each IPFP tissue sample contains a fair number of human perivascular stem cells (hPSCs) or human mesenchymal stem cells (hMSCs), irrespective of donor age and gender

**Fig. 1:**
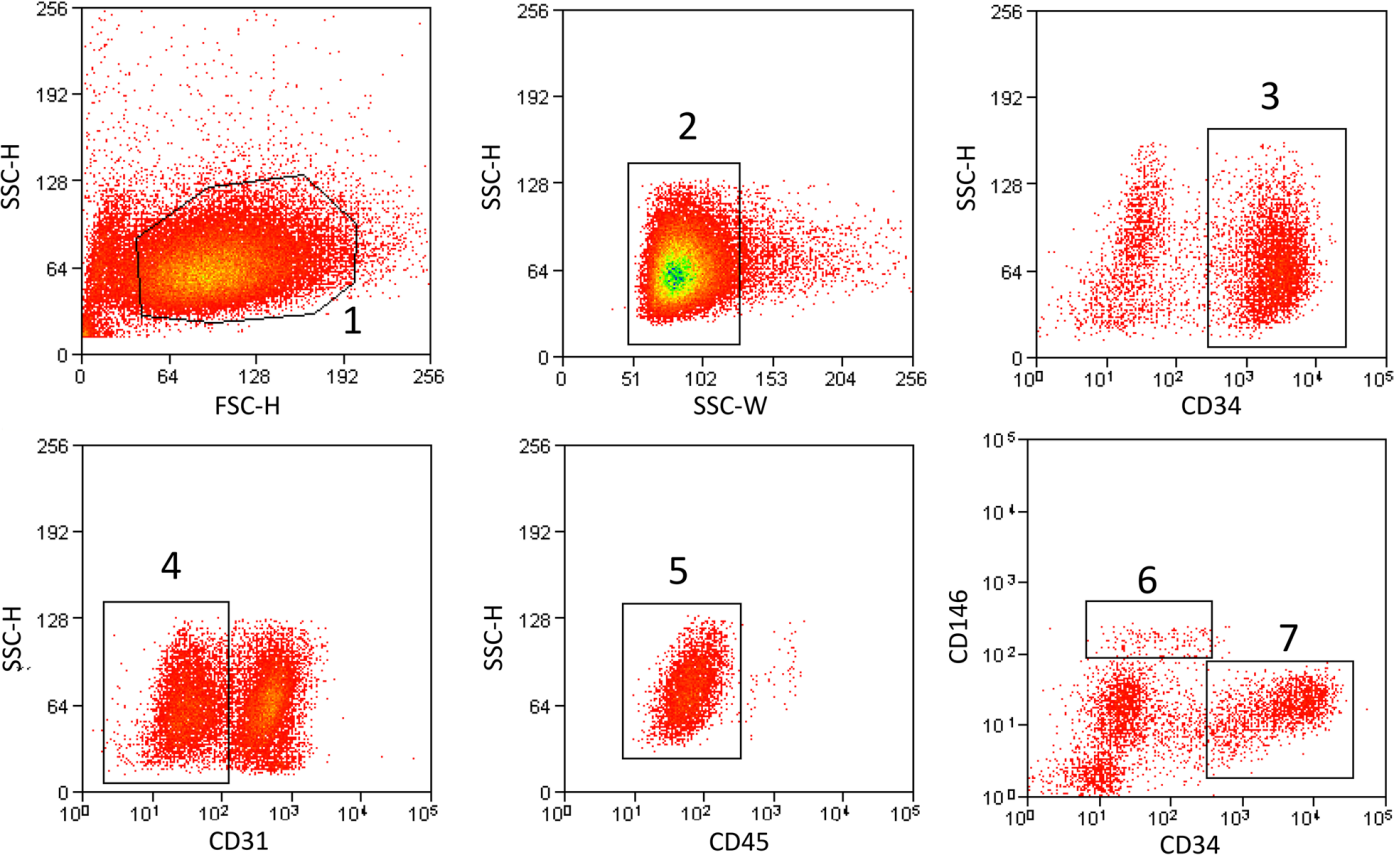
Gating strategies for the isolation of Adipose-Derived Mesenchymal Stem Cells (ADMSCs) from the infrapatellar fat pad (IPFP). Gate 1 = cells, Gate 2 = single cells, Gate 3 = CD31^−^ cells, Gate 4 = CD31^−^, CD45- cells (to remove hematopoietic cells and endothelial cells), Gate 5 = pericytes (CD34^−^, CD31^−^, CD45-, CD146^+^) and Gate 6 = adventitial cells (CD34^+^, CD31^−^, CD45^−^, CD146^−^). FSC: forward scatter, SSC: side scatter

The IPFP was isolated from total knee arthroplasties in 96 patients and was digested by collagenase for stromal vascular fraction (SVF) isolation. Demographic information as well as sample statistics, cell yield, viability, and subpopulation statistics have been summarized for 96 unique donor samples (Table 2), of which 70 were female donors and 26 were male, and having a mean age of 58 years (range 15–82) and a mean BMI of 25.16 (range 18.07–36.67). The mean yield of cells was 2.63 × 10^6^ per 7.08 g of human IPFP (range 0.05 × 10^6^–10.3 × 10^6^, *n* = 96). The mean viability of freshly isolated SVF cells was 85.16% (range 70.5–95.6%). Perivascular stem cells (PSCs) are cell populations that are comprised of pericytes (CD31^−^ CD45^−^ CD34^−^ CD146^+^) and adventitial cells (CD31^−^ CD45^−^ CD34^+^ CD146^−^), which are a homogeneous MSC population purified by fluorescence activated cell sorting (FACS) [42, 45–47]. Pericytes and adventitial cells have been reported to possess characteristics of MSCs [45, 48]. MSC cells were purified from the isolated SVF by FACS using the gating parameters shown in Fig. 1. FSC-Height and SSC-Height were used first for gating debris-free cells from cell suspensions made from IPFP. Then, SSC-Height and SSC-Width were used for gating single cells. CD45^−^ hematopoietic cells and CD31^−^ endothelial cells averaged 47.85% (range 14.69–77.88%; *n* = 52) of total live cells in the SVF. The primary MSCs were purified by CD34 and CD146 according to previously published methods [42, 48]. The mean proportion of MSCs was 51.61% (range 1.99–87.4%; *n* = 52) with a mean proportion of pericytes of 4.51% (CD31^−^, CD45^−^, CD34^−^, CD146^+^, range: 0.21–20.31%; *n* = 52), and adventitial cells of 47.1% (CD31^−^, CD45^−^, CD34^+^, CD146^−^, range 1.07–82.44%; *n* = 52). This represents a mean yield of 1.52 × 10^6^ MSCs per 7.08 g of human IPFP (range 7 × 10^3^–6.48 × 10^6^; *n* = 52).

**Table 2:** Demographic data of the 96 donors

Data	
Sex	Female = 70
	Male = 26
Age (years)	Mean = 58 (range 15–82)
Volume (ml)	Mean = 4.1 (range 0.5–15)
Weight (g)	Mean = 7.1 (range 1.2–20.3)
BMI (Kg/m^2^)	Mean = 25.2 (range 18.1–36.7)
SVF (cells × 10^6^)	Mean = 2.6 (range 0.05–10.3)
Viability (%)	Mean = 85.2 (range 70.5–95.6)
Pericytes (%)	Mean = 4.5 (range 0.2–20.3)
Adventitial cells (%)	Mean = 47.1 (range 1.1–82.4)
MSCs total (%)	Mean = 51.6 (range 2.0–87.4)
MSCs yield (cells × 10^6^)	Mean = 1.5 (range 0.08–6.5)

*BMI* body mass index, *SVF* stromal vascular fraction, *MSCs* mesenchymal stem cells

**Fig. 2:**
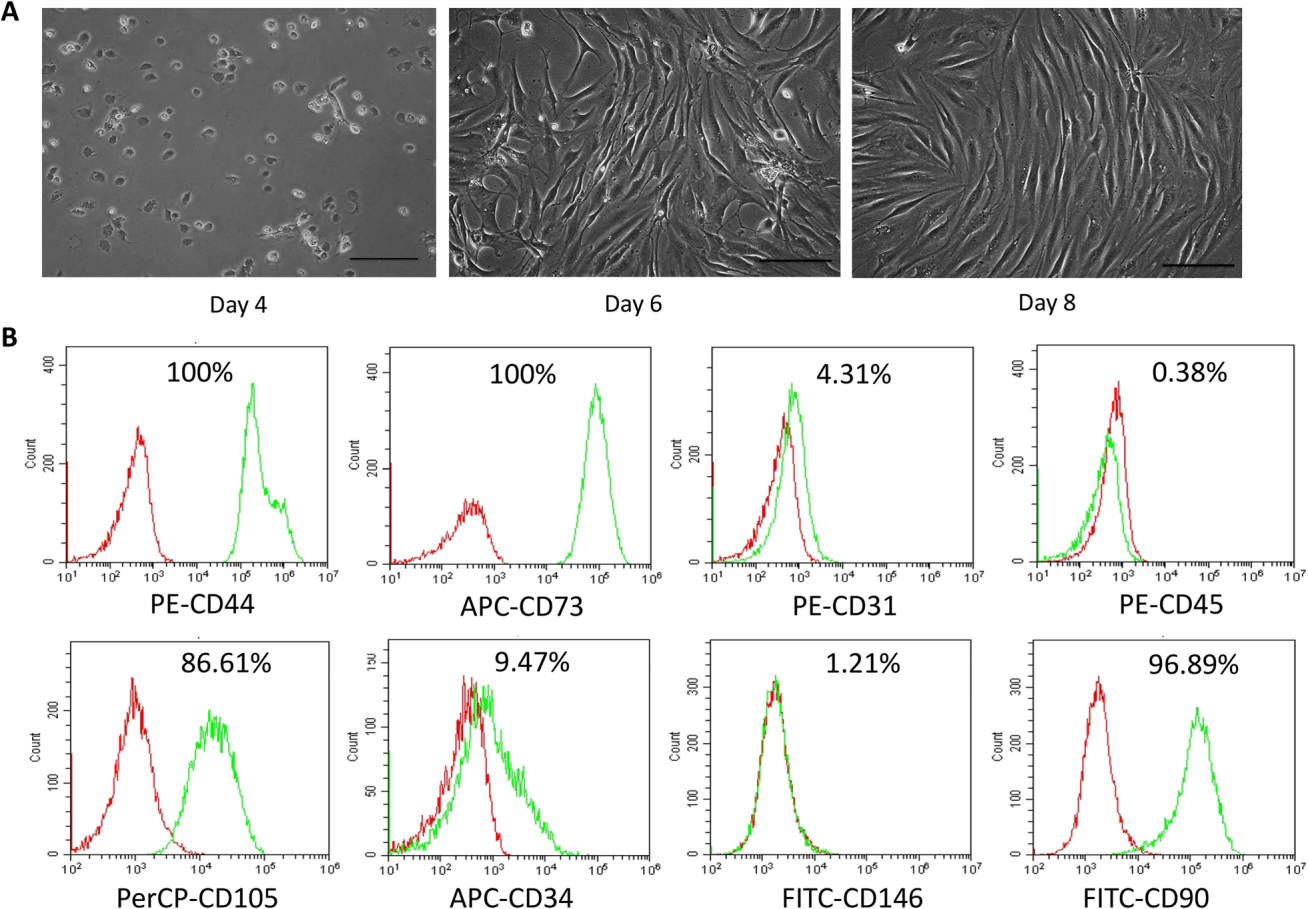
The microscopic appearance and surface markers of ADMSCs isolated from the infrapatellar fat pad (IPFP). **A** ADMSCs isolated from the IPFP gradually showed a typical spindle-shaped morphology. Scale bar: 100 μm. **B** ADMSCs isolated from the IPFP were positive for mesenchymal stem cell markers (CD44, CD73, CD90, and CD105) and negative for hematopoietic marker (CD45) or endothelial marker (CD31)

Once the adipose tissue-derived mesenchymal stem cells are expanded in culture, the expression of their surface markers gradually changes [49]. The purified MSCs displayed a spindle-shaped morphology typical of MSCs after culture at day 8 (Fig. 2A). Flow cytometry analysis (Fig. 2B) showed that the cultured MSCs were positive for the surface markers CD90 (96.89%), CD105 (86.61%), CD73 (100%), and CD44 (100%), but negative for CD31 (4.31%) and CD45 (0.38%) at passage 6. Subsequent to the cultures, the expression of CD34 (9.47%) and CD146 (1.21%) was decreased at passage 6.

### Higher yields of hMSCs from IPFP harvests having significant inflammation

**Fig. 3:**
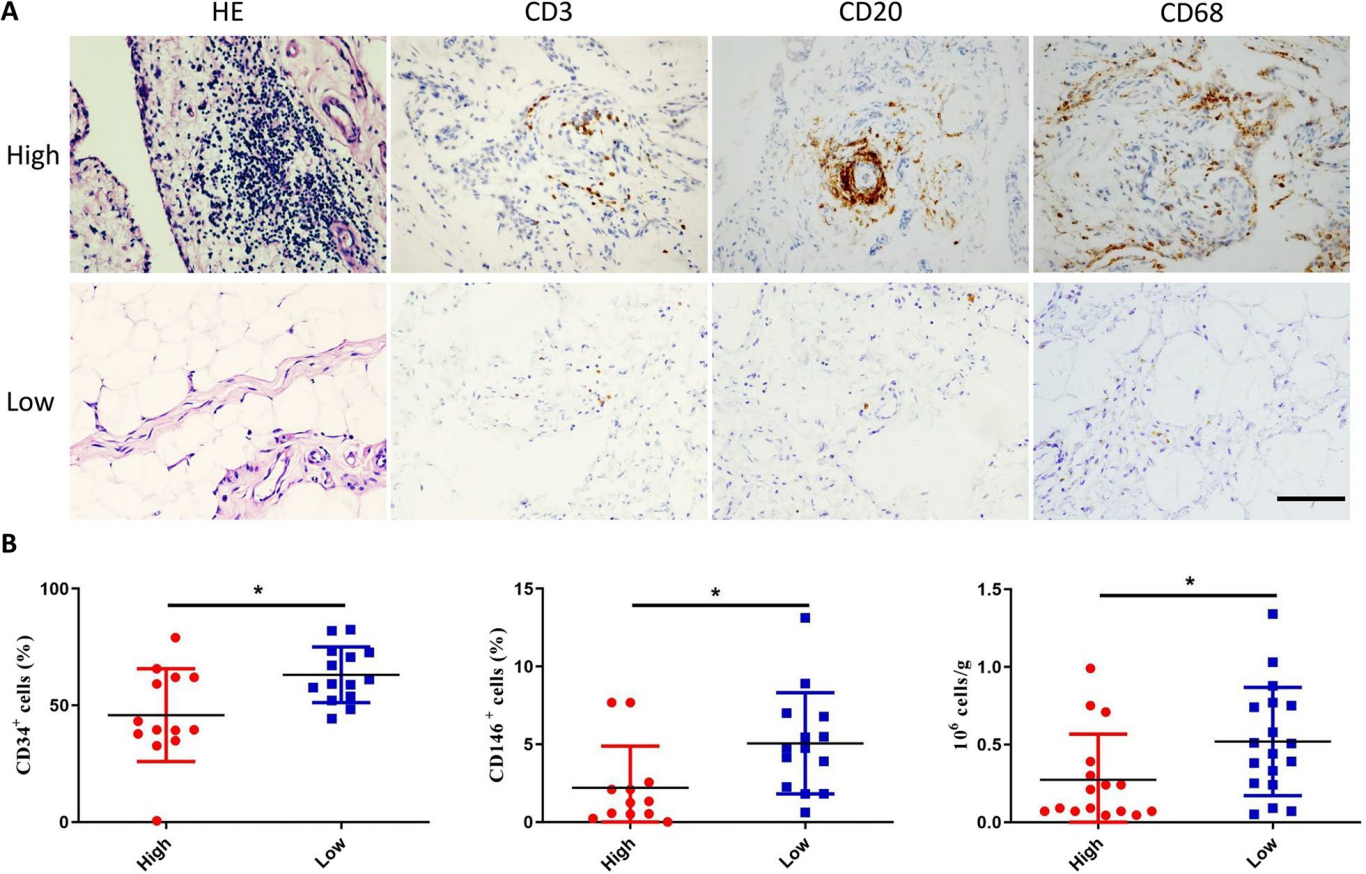
Effect of degree of inflammation on SVF/PSC yields. **A** HE staining and immunohistochemical staining of CD3 (T-cells), CD20 (B-cells) and CD68 (macrophages) were performed on infrapatellar fat pad tissue samples (*n* = 31; male = 20, female = 11). High: the IPFP tissues with high inflammation level (positive in CD3, CD20, and CD68 staining). Low: the IPFP tissues with low inflammation level (negative in CD3, CD20, and CD68 staining). Scale bar: 100 μm. **B** Bar chart showing the mean ± standard deviation for the CD34^+^ cells, CD146^+^ cells, and total number of ADMSCs isolated from the IPFP tissues with high and low inflammation levels. Statistically significant differences are indicated (*p < 0.05)

To understand whether inflammatory severity inherent in IPFPs has any impact on production of MSCs, small portions of tissue samples from extracted MSCs were fixed with 4% paraformaldehyde and then dehydrated, embedded, and sectioned for immunohistochemical and HE staining for CD3, CD20, and CD68. Notably, lymphocytes and macrophages were the predominant cells of inflammation. T-cells and B-cells express CD3 and CD20 [50, 51], respectively, and macrophages express CD68 [52]. Inflammatory cells have large nuclei and large nucleoplasmic ratios. The nuclei are distinctly blue after HE staining, and the cytoplasm is pink to peachy in color. Tissues with a high degree of inflammation highly expressed CD3, CD20, and CD68, and there were many inflammatory cells seen on HE staining (Fig. 3A). Based on semi-quantitative analysis, we determined that the tissues with more than 20 CD68-positive macrophages/mm^2^ of tissue as the high inflammation group, and tissues with less than 20 CD68-positive macrophages/mm^2^ of tissue as the low inflammation group (Additional file 1: Fig. S1). Thus, MSCs derived from highly inflamed tissues were defined as High-MSCs, and MSCs derived from lowly inflamed tissues were defined as Low-MSCs. After statistical comparison, the percentage of CD34^+^ and CD146^+^ expression levels and total cell acquisition rates were found to be statistically significantly different between cells of highly and lowly inflamed tissue origin (*p* < 0.05*). The percentage of CD34^+^ and CD146^+^ cell acquisition was higher in the lowly inflamed tissue-derived cells (High group: CD34^+^: 32.67–82.44%, mean 51.3%; CD146^+^: 0–20.31%, mean 4.23%. Low group: CD34^+^: 44.32–73.28%, mean 61.19%; CD146^+^: 0.62–13.13%, mean: 5.79%). Total cell acquisition rate was also higher in the lowly inflamed tissue-derived cells (High group: 0.04 × 10^6^–1.34 × 10^6^, mean 0.38 × 10^6^; Low group: 0.046 × 10^6^–0.87 × 10^6^, mean 0.42 × 10^6^) (Fig. 3B). This suggests that source tissue inflammatory status influences the acquisition rate of MSCs, and the expression of CD34 and CD146.

### Trilineage differentiation potential of MSCs may not affected by IPFP inflammation

**Fig. 4:**
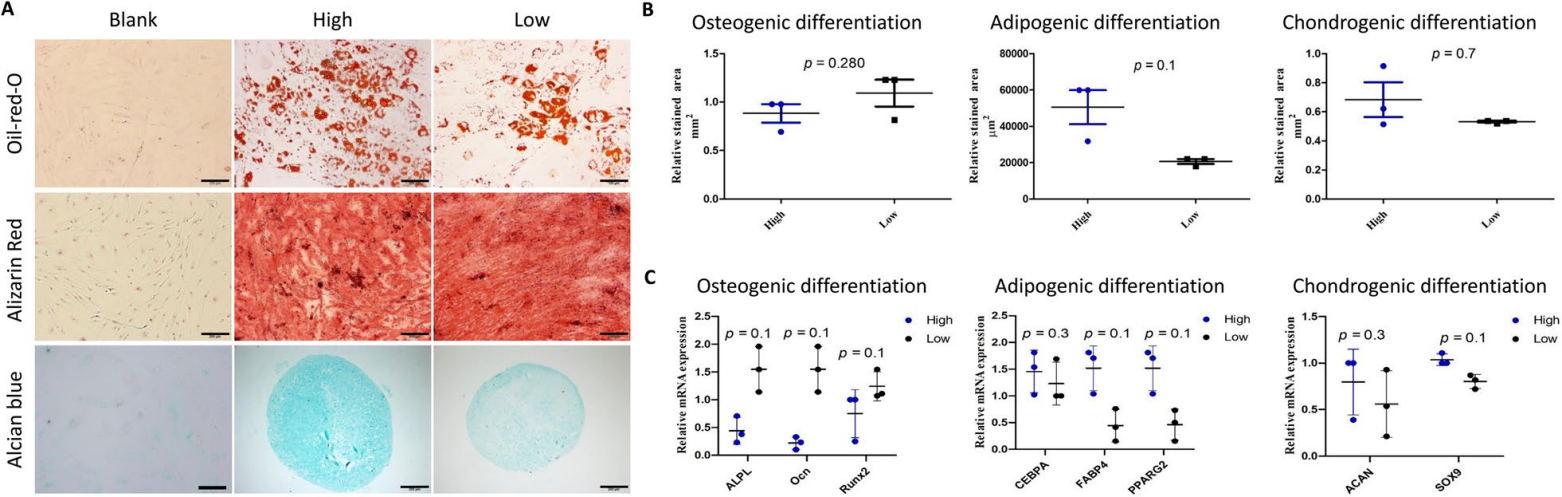
Effect of degree of inflammation on trilineage differentiation potential of ADMSCs. High: ADMSCs isolated from IPFP with high inflammation level; Low: ADMSCs isolated from IPFP with low inflammation level. **A** Representative images of adipocyte, chondrocyte, and osteocyte differentiation of ADMSCs isolated from the high (*n* = 3; male = 2, female = 1) and low inflammation IPFP tissues (*n* = 3; male = 1, female = 2). Adipogenic differentiation was assessed by Oil Red O staining, osteogenic differentiation was assessed by Alizarin Red staining, and chondrogenic differentiation was assessed by Alcian blue staining. Scale bar: 200 μm. **B** Quantitative analysis of the stained areas with Oil Red O, Alizarin Red, and Alcian blue. **C** The expression levels of chondrogenic marker genes (ALPL, Ocn, Runx2), adipogenic marker genes (CEBPA, FABP4, PPARG2), and chondrogenic marker genes (ACAN, Sox9) measured by qRT-PCR

Under appropriate stimulation and culture conditions, MSCs have the capacity to differentiate into adipocytes, chondrocytes, and osteoblasts. Our results were obtained via directional staining and qRT-PCR. For adipogenic differentiation, Oil red O staining showed that a large number of round lipid droplets were visible in cells after 14 days of MSC-induced adipogenic differentiation. For osteogenic differentiation, Alizarin Red Staining showed that MSC-induced osteogenic differentiation formed obvious mineralized nodes, which stained orange. For chondrogenic differentiation, Alcian blue binding assay showed that MSC-induced chondrogenic differentiation formed chondrospheres after 21 days (Fig. 4A). Statistical analysis of the staining results showed that MSCs derived from highly inflamed tissue were more capable of chondrogenic and adipogenic differentiation, while MSCs derived from lowly inflamed tissue were more capable of osteogenic differentiation (Fig. 4B). In addition, we examined the expression of the chondrogenic genes, ALPL, Ocn, and Runx2, the adipogenic genes, CEBPA, FABP4, and PPARG2, and the chondrogenic genes, Acan and Sox9 (Fig. 4C). The MSCs from high-inflammation tissue have higher adipogenic and chondrogenic and lower osteogenic gene expressions, while the in vitro cell differentiation data exhibited the same trend. However, neither of them shows statistical significance. This indicates that source tissue inflammatory status may have minimal or no impact on the differentiation capacity of MSCs.

### Clonogenic capacity of MSCs is independent of the inflammation status of the IPFP

**Fig. 5:**
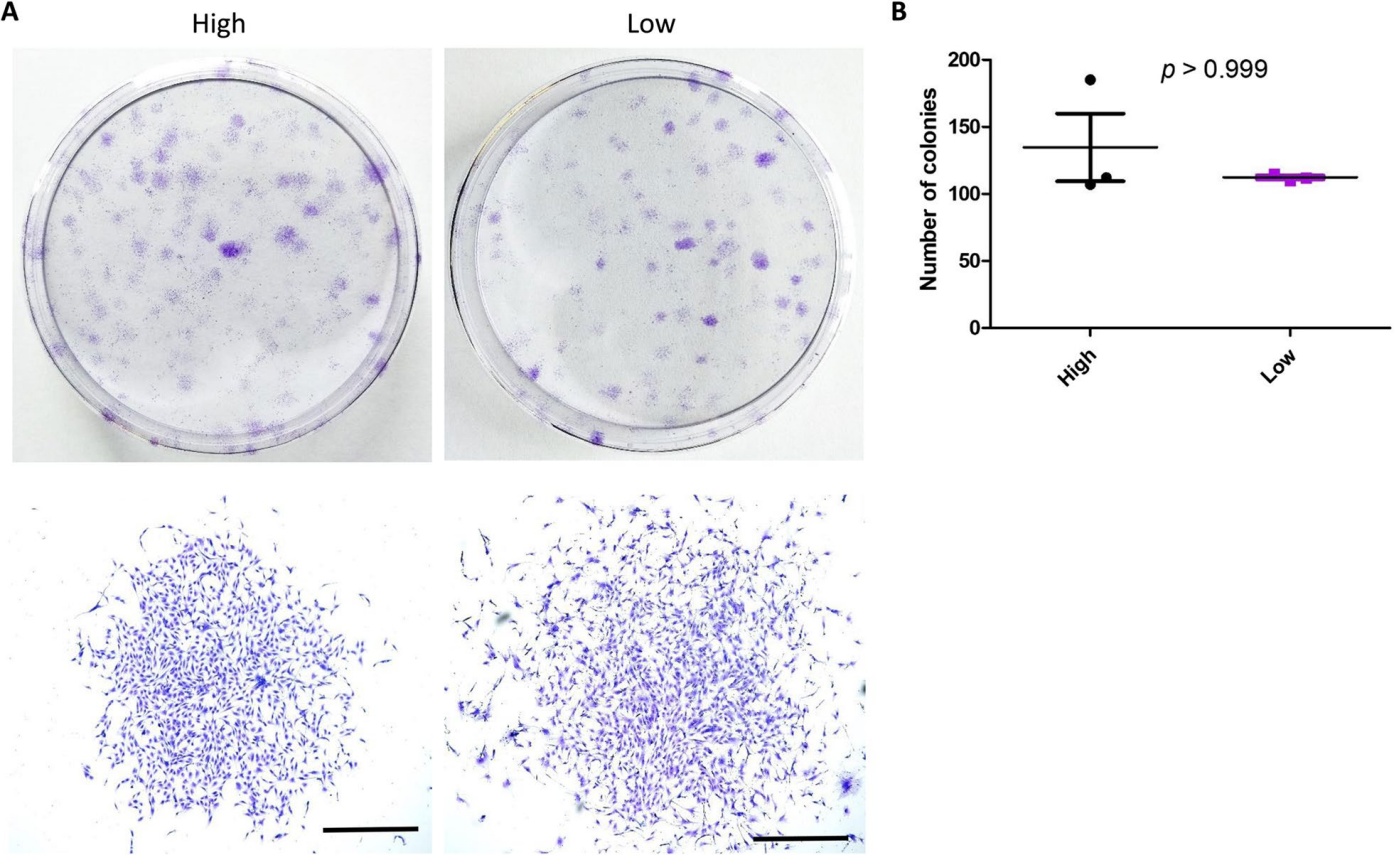
Effect of inflammation on the clonogenic capacity of ADMSCs. **A** Colony formation of ADMSCs isolated from the high (*n* = 3; male = 2, female = 1) and low inflammation IPFP tissues (*n* = 3; male = 1, female = 2). Individual colonies are shown in the lower panel. Scale bar = 1 mm. **B** The quantitative analysis of the number of colonies

To examine the effect of tissue inflammation on the proliferative capacity of MSCs, 200 MSCs each of highly inflammatory tissue origin and of lowly inflamed tissue origin were inoculated into culture dishes. Clones were formed in both groups of MSCs after 14 days of culture. After paraformaldehyde fixation, single cell-formed clones were observed by crystal violet staining (Fig. 5A). By counting the number of clones formed, it was observed that the clonogenic capacity of MSCs of highly inflamed tissue origin was higher than that of MSCs of lowly inflamed tissue origin; however, this difference in clonogenic capacity was calculated to not be statistically significant (number of clones formed in the highly inflamed group: 107–185, mean 135; number of clones formed in the lowly inflamed group: 110–115, mean 112) (Fig. 5B). This indicates that the intensity of source tissue inflammation in the IPFP does not significantly impact on the proliferative capacity of MSCs.

### The transcriptomics of MSCs remains relatively stable in both highly and lowly inflamed IPFP tissues

**Fig. 6:**
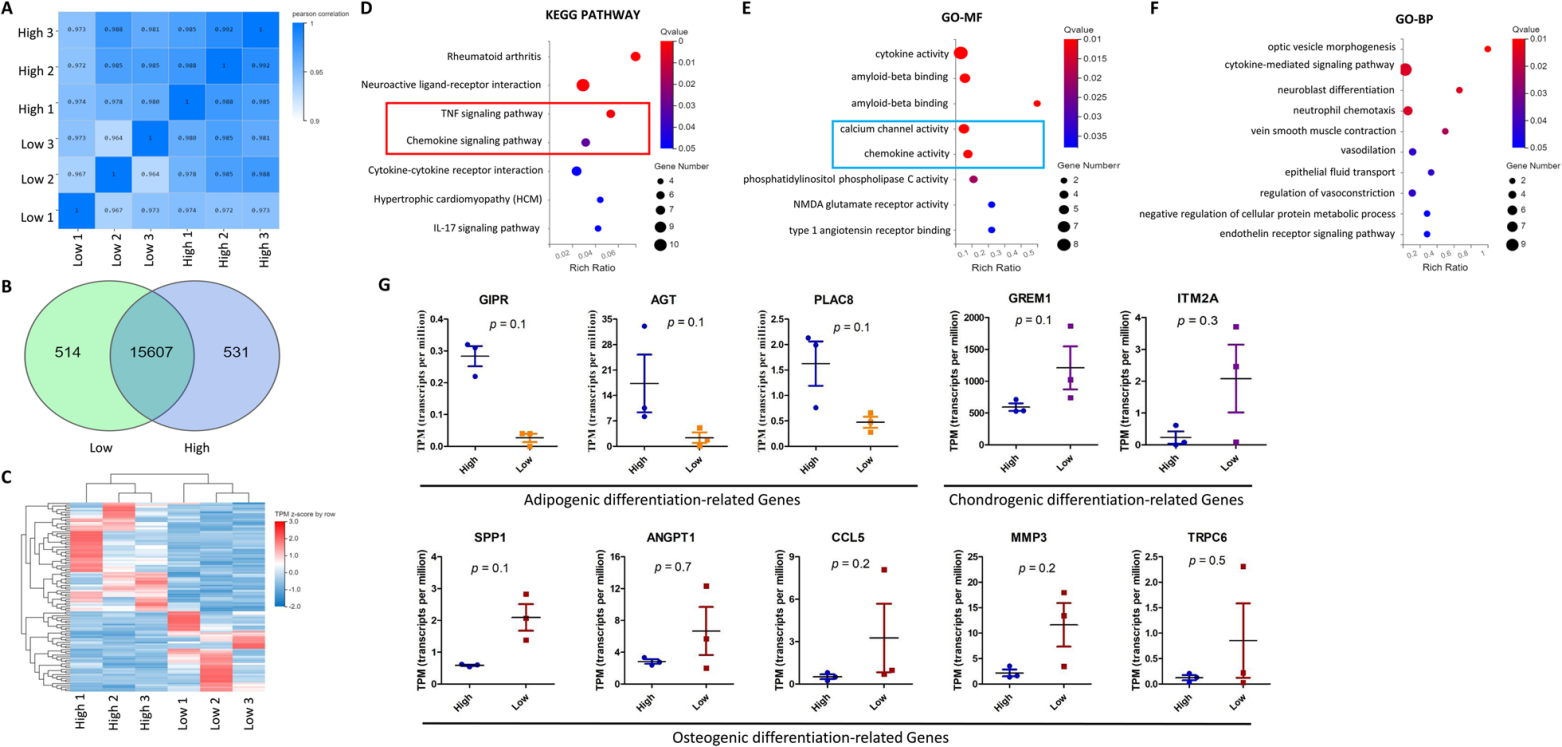
Effect of inflammation on Gene Expression of ADMSCs. **A** Correlation of expression patterns in ADMSCs between high (*n* = 3; male = 2, female = 1) and low (*n* = 3; male = 1, female = 2) inflammation level. Correlation matrix of the entire dataset. The analysis was performed by comparing the values of the entire transcriptome in all six samples. Correlation analysis was performed using R software. **B** Venn chart for gene expression comparisons between High (blue) and Low (green) inflammation level. **C** Heat map of 106 differentially expressed genes in ADMSCs from High versus Low inflammation level. Each row represents a gene. Expression values for each gene are scaled across each row as *z*-scores. *n* = 3 for each group. **D** KEGG pathway database was used for analyzing differentially expressed genes. Seven signaling pathways were significantly enriched in ADMSCs from the high inflammation tissue, and two of the pathways (red box) were involved in regulating the adipogenic differentiation of MSCs. **E**, **F** Gene Ontology (GO) enrichment analysis of 106 differentially expressed genes. Bar graph shows fold enrichment for the top five GO Biological Process terms enriched at *p* ≤ 0.05. MSC adipogenic differentiation-related pathways were significantly enriched in High-MSC group cells (blue box). GO-MF (e), GO Molecular function; GO-BP (f), GO Biological process. **G** Differentially expressed marker genes related to the MSC trilineage differentiation. The gene expression levels are estimated in terms of “transcripts per million”

MSCs have been recognized as promising candidates for cellular therapy and are broadly used in clinical trials [28]. A large number of bioactive compounds (cytokines, growth factors, antiapoptotic and antioxidative factors, extracellular matrix components, extracellular vesicles) secreted by MSCs are activated in response to tissue injury to promote regeneration and restore physiological homeostasis. These compounds are major components in the coordination of regenerative processes associated with tissue healing [53, 54]. In order to improve the therapeutic potential and safety of MSC use in clinical treatment, we analyzed the gene expression profiles of MSCs derived from varying degrees of source tissue inflammation. We selected three primary MSCs each from highly inflamed tissue origin and lowly inflamed tissue origin as independent replicates from the sample (*n* = 3). RNA-sequencing identified global changes in gene expression in MSCs from different source levels of tissue inflammation. A total of six primary MSCs were measured using the DNBSEQ platform, and each sample yielded an average of 6.65 gigabytes of data. The target genomes were matched at 95.22%, and the average gene set was 83.59%, with a total of 16,652 genes being detected. To reflect the correlation of gene expression between samples, the Pearson correlation coefficients of all gene expressions between each of two different samples were calculated, and these coefficients were reflected in the form of heat maps. The correlation coefficients reflect the similarity of overall gene expression between the individual samples, and the higher the correlation coefficient, the more similar the gene expression levels are. Our correlation analysis showed that our calculated Pearson correlation coefficients were all greater than 0.95, and close to 1, suggesting that the six primary MSCs were highly correlated, with very little variability (Fig. 6A). The boxplot shows the distribution of gene expression levels in each sample, and it can be observed that the dispersion of the data distribution is also quite uniform (Additional file 2: Fig. S2A). Just one differentially expressed gene was found to be upregulated in MSCs derived from highly inflamed IPFPs (Additional file 2: Fig. S2B), as determined by |log2(fold-change)|> 1 and *p* < 0.05. The Wayne diagram showed that highly inflamed tissue-derived MSCs and lowly inflamed tissue-derived MSCs co-expressed a total of 15,607 genes, of which 514 genes were specifically expressed in highly inflamed tissue-derived MSCs and 531 genes were specifically expressed in lowly inflamed tissue-derived MSCs (Fig. 6B). We analyzed the transcriptome sequencing results online using Dr. Tom, and a total of one hundred and six differentially expressed genes (fold change > 2.0 and *p* < 0.05) were screened out between the populations of highly inflamed tissue-derived MSCs and lowly inflamed tissue-derived MSCs, and there were 45 upregulated and 61 downregulated genes in the lowly inflamed tissue-derived MSCs (Additional file 3: Tables S1, S2). Figure 6C shows the expression heat map of these differentially expressed genes in every sample. To further analyze the potential mechanism of the effect of degree of tissue inflammation on the trilineage differentiation potential of MSCs, we performed GO and KEGG pathway enrichment analysis on differentially expressed genes from RNA-sequencing data. The enrichment analysis was performed using the ‘phyper’ function in R software according to the KEGG pathway annotation classification. The *p* values were calculated, and the *q*-value was subsequently obtained by FDR correction of the *p* values, and a function with *p* ≤ 0.05 was considered significantly enriched. The KEGG enrichment results showed that MSC cells of highly inflamed tissue origin were significantly enriched in seven signaling pathways, among which the TNF signaling pathway and the chemokine signaling pathway (red box), which are related to adipogenic differentiation of MSC cells, were more significantly altered (Fig. 6D). The results of GO analysis also showed that adipogenic differentiation-related pathways (calcium channel activity and chemokine activity) were significantly enriched in the highly inflamed tissue-derived MSC group of cells (blue box) (Fig. 6E). The GIPR, AGT, and PLAC8 genes are related to the positive regulation of adipogenic differentiation; they indeed show significantly higher expression in MSCs of highly inflamed tissue origin. In comparison, the GREM1 and ITM2A genes that play a role in chondrogenic differentiation and the SSPP1, ANGPT1, CCL5, MMP3, and TRPC6 genes that play a role in osteogenic differentiation were found to be upregulated in MSCs derived from lowly inflamed tissue (Fig. 6G). Biological processes related to the differentially expressed genes were also identified. The optic vesicle morphogenesis pathway, the cytokine-mediated signaling pathway, the neuroblast differentiation pathway, and the neutrophil chemotaxis pathway are the most activated pathways in highly inflamed tissue-derived MSCs (Fig. 6F). These observations indicate that MSCs of lowly and highly inflamed tissue origin have a distinct transcriptional regulation at basal levels. Highly inflamed tissue-derived MSCs are characterized by the upregulation of adipogenic differentiation processes, and the cytokine-mediated signaling pathway was enriched, which may be involved in the regulation of cell growth and differentiation, immune functions, and in processes such as inflammation development and damage repair.

### ***Inflammation levels of tissue of origin influences regenerative efficiency of MSCs in a CCl***_***4***_***-induced liver injury model***

**Fig. 7:**
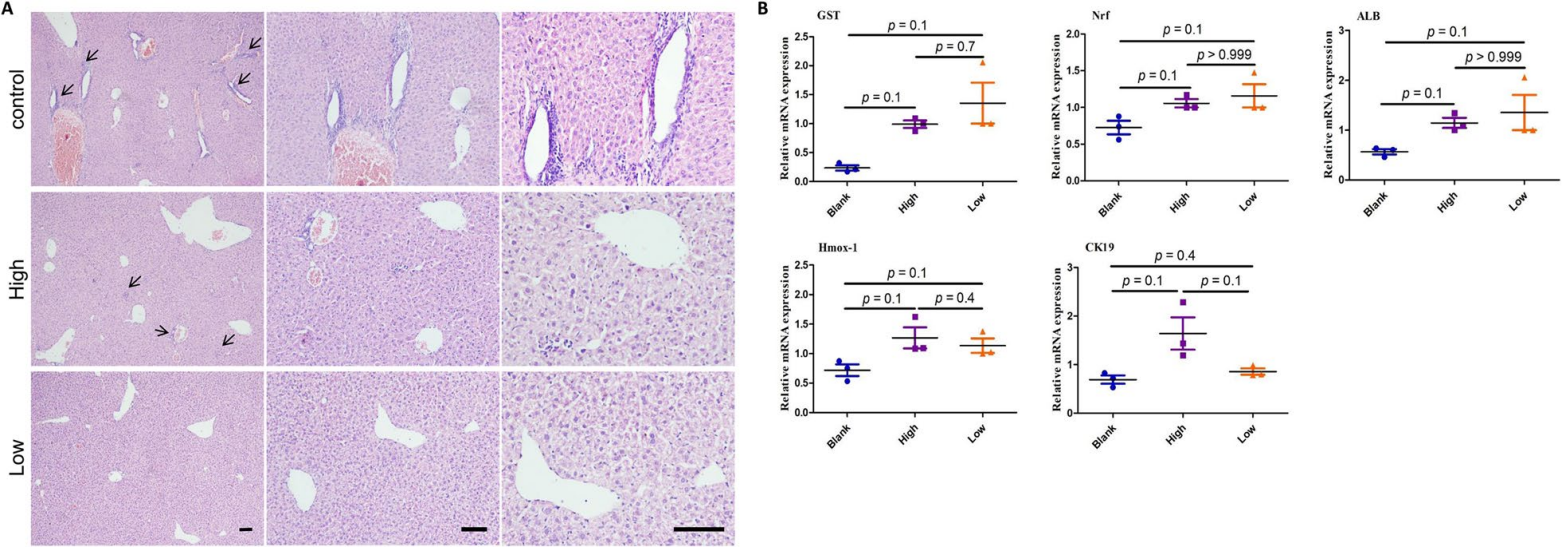
Effect of the inflammation on the regenerative ability of ADMSCs by using a CCl_4_-induced liver injury murine model. **A** HE staining of liver tissue sections. Control: transplanted with PBS; High: transplanted with ADMSCs from high inflammation IPFP tissue; Low: transplanted with ADMSCs from low inflammation IPFP tissue. The hepatic necrosis around the vessels are shown by arrows. Scale bar = 100 µm. **B** Expression levels of liver function-related regulatory genes (ALB, Nrf, GST, Hmox-1, CK19) were measured by qRT-PCR

MSCs have potential for tissue repair and regeneration. Subsequent to being expanded, cultured, and transplanted into damaged tissues, they modulate inflammatory responses by synergistically down-regulating pro-inflammatory cytokines and up-regulating anti-inflammatory cytokines [55]. However, safety of MSCs in regenerative therapy needs to be well evaluated. To examine the effect of the degree of inflammation of the source tissue on the tissue repair ability and safety of MSCs, we transplanted MSCs sourced from IPFP tissue having different degrees of inflammation and PBS (control group), by tail vein injection into mice with acute liver injury induced by two consecutive days of CCl_4_ injection. Mice were euthanized after 14 days and their livers were harvested. Morphological changes in injured liver tissue in each group were observed by HE staining of tissue sections (Fig. 7A) and qRT-PCR was performed to detect the expression of genes related to liver function (Fig. 7B).

The normal liver is lobular with a clear structure, and consisting of uniformly sized hepatocytes arranged in patches, with a central vein in each lobule and hepatocytic plates radiating from the vein to the periphery. In livers of mice after injection with CCl_4_, there was damage to hepatocytes in the periportal and central regions, with marked cellular edema and diffuse inflammatory cell infiltration of the liver tissue. As shown in Fig. 7A, after transplantation of MSCs both groups (lowly inflamed tissue-derived MSCs and highly inflamed tissue-derived MSCs) showed restoration of hepatic lobular structures and reduction of paracentral necrosis, compared to the control group (PBS). Albumin (ALB) is synthesized by the liver and is an important indicator of the synthetic functioning of the liver. Nrf-2, GST, and Hmox-1 are cytoprotective molecules and play an important role in detoxification. qRT-PCR results on liver tissues showed that the expression of ALB, Nrf-2, GST, and Hmox-1 was higher in the treated group, compared with the control group. This indicates partial restoration of liver function after MSC treatment, with significant changes related to ALB, Nrf-2, and GST. However, we observed no mathematically significant difference between the lowly and highly inflamed tissue-derived MSC groups.

## Discussion

Inflammation is an important factor which affects the functions of adult stem cells in vivo and can affect the proliferative and differentiation capability of adult stem cells. Studying the effects of inflammation on the proliferation and differentiation ability of adipose-derived MSCs may establish a theoretical foundation for autologous tissue repair. The cellular microenvironment surrounding MSCs, especially inflammatory factors, play a crucial role in their self-renewal, proliferation and differentiation, and dysfunction of microenvironmental factors can cause enhanced or diminished MSC function [56, 57]. Alongi et al. selected four markers, i.e., STRO-1, CD90, CD105, and CD146 to identify MSCs. Immunofluorescence staining revealed that the density of the four markers was significantly higher in inflamed pulp than in normal pulp, which may be due to increased angiogenesis in the inflamed tissue, as the preceding markers are associated with blood vessels [58]. However, it has also been shown that the inflamed microenvironment does not affect the surface markers of stem cell [59]. The results of our study observed that tissue inflammation status does not affect the expression of surface markers of MSCs.

It has been suggested that excessive proliferation of inflammatory cells is both a pathological feature of chronic inflammation and an initiating factor in its pathogenesis. Stem cell proliferation is in a relatively quiescent state under normal conditions, and when stimulated by inflammatory factors, stem cells enter a transient expansion state. In the present study, MSCs were obtained from different inflammatory fat pad samples, and the primary cells were found to grow adhering to the cell culture dish, with spindle-shaped morphology and large nuclei. The cells were analyzed by flow cytometry analysis using surface molecular markers of MSCs and showed high expression of CD44, CD73, CD105, and CD90.

Inflammation is a complex process, and depending on the degree of inflammatory stimulation, different stimulators may cross react with each other to regulate certain properties of MSCs. Many studies have shown that the Wnt pathway is closely related to the stemness of stem cells [60, 61]. A large number of LEF-β-catenin complexes are formed to activate the target gene Cyclin D1 when the Wnt/β-catenin pathway is activated. This regulates the cell growth cycle to promote stem cell proliferation, which may explain the increased proliferative capacity of MSCs in inflamed environments [62]. The ability of colony formation may reflect the proliferative capacity of MSCs. However, our results have shown that the baseline inflammatory level of the tissue of origin does not affect colony formation of MSCs (Fig. 5A, B). This indicates that the proliferative capacity of MSCs may likely be influenced by other factors.

It has been demonstrated that inflammatory factors such as tumor necrosis factor-alpha (TNF-α) in the inflamed microenvironment may activate the Wnt pathway and cause a decrease in the differentiation capacity of MSCs, while inhibition of the Wnt pathway could modulate the effect of inflammation on osteogenic and lipogenic differentiation capacity. Periodontal ligament stem cells in an inflamed environment may have their osteogenic capacity influenced by inhibition of the non-classical Wnt/Ca^+^ signaling pathway [58]. TNF-α production, induced by lipopolysaccharide release in periodontitis, significantly inhibits osteogenic differentiation of periodontal ligament stem cells, resulting in the loss of periodontal tissue regenerative capacity. TNF-α plays an important role in bone pathophysiology by inhibition of transcriptional regulation of Runx2 expression [58, 63–65], and it has also been shown that TNF-α inhibits osteogenic differentiation and the formation of mineralized nodules [66]. These facts suggest that the secretion of inflammatory factors may affect the biological characteristics of stem cells through a variety of complex signaling mechanisms [67, 68]. In the present study, MSCs differentiated toward adipocytes, osteoblasts, and chondrocytes under specific conditions, when comparing the effects of different degrees of inflammation on the multidirectional differentiation capability of source MSCs. Our results indicated that although there was no significant difference between the two groups, but there are more lipid droplets were stained with Oil red O in MSCs sourced from IPFPs with higher degrees of inflammation than in MSCs sourced from IPFPs with lower degrees of inflammation, and the cartilage ball formed by MSCs sourced from IPFPs with higher degrees of inflammation were larger than in MSCs sourced from IPFPs with lower degrees of inflammation, indicating that inflammation may promote the differentiation of MSCs toward adipocyte and chondrocyte cells. Fewer mineral nodules were stained red in MSCs sourced from IPFPs with a higher degree of inflammation than in MSCs sourced from IPFPs with a lower degree of inflammation, indicating that inflammation inhibits the osteogenic differentiation of MSCs. This suggests that tissue inflammation may affect the differentiation capacity of MSCs to some extent, which may be due to the presence of various inflammatory transmitters in the inflammatory microenvironment, such as inflammatory cells, inflammatory factors, and leukocyte metabolites inducing continuous stimulation of MSCs, thus altering their proliferation and differentiation capacity.

Among the cohort of 96 donors, the majority lacked documented comorbidities in their medical records. Three donors exhibited hypertension, while two others had diabetes. It is known that hypertension can trigger a systemic pro-inflammatory state. Exploring whether this state could further impact MSC performance holds promise for future investigations. Concerns have been raised about potential MSC dysfunction in donors with comorbidities. However, recent research has demonstrated that donor demographics and the presence of comorbidities like diabetes mellitus, coronary artery disease, rheumatoid arthritis, and systemic immunosuppression exert limited influence on MSC osteogenic potential [69]. Undoubtedly, to ensure the safety profile of MSCs derived from patients with comorbidities, a more comprehensive inquiry into tri-lineage differentiation attributes, reparative capabilities, and other pertinent factors is imperative.

Two clinical trials have been performed in patients with liver cirrhosis and acute or chronic liver failure, and results from these trials indicate that huMSCs have good clinical efficacy, and induces improved liver function and an increased survival rate [70, 71]. In recent years, it has been shown that the mechanism of action of MSCs in tissue repair may involve secretion of soluble factors that alter the tissue microenvironment [72]. Acute tissue injury is usually accompanied by inflammation and cellular necrosis. Cytokines are released from cells, and microvascular damage leads to increased vascular permeability, macrophage and neutrophil infiltration, and the phagocytosis of necrotic cells results in the release of proinflammatory mediators such as interferon γ, tumor necrosis factor α, interleukin 1, chemokines, leukotrienes, and free radicals. MSCs are able to resist damage signals and potency to be specifically activated in response to tissue injury to promote tissue regeneration through different mechanisms. In addition, MSCs may recruit functional cells into a stem cell niche or differentiate into the missing cell components, and the stem cell niche in turn regulates the fate of stem cells [73]. CCl_4_-treated mice are often used as an experimental model for studying hepatic fibrosis. After two consecutive days of once per day injection of CCl_4_, we successfully established a mouse model of acute liver injury. Our data indicate that MSCs obtained from differing source degrees of inflammation can restore some liver functions and morphology in mice. Changes in the tissue microenvironment during the pathogenesis and regression of liver disease are multifarious, and it is therefore necessary to explore mechanisms whereby underlying tissue inflammation exerts differing regenerative effects on MSCs in liver injury development and healing.

Anatomical location, relative accessibility, and minimal risk of complications make the IPFP a high quality source of stem cells for regenerative medicine [74]. Several limitations exist, however, to discourage further study of potential clinical applications for IPFP. Studies have traditionally used IPFP tissue from patients undergoing knee surgery, which may have severe disease and thus differ from IPFP tissue from healthy patients. Stem cell quantity and quality may also vary with age, which may potentially limit the autologous use of stem cells in older patients. Additionally, different types of inflammation induced by either infection, degenerative disease, neoplastic disease, or trauma in the source IPFP may result in variations in the IPFP derived stem cell population. Thus, inflammation may limit the clinical application of stem cells. Further studies are needed to confirm differences in IPFP mesenchymal stem cells between healthy and injured/diseased tissues, and among donors of different ages. The present study provides a knowledge base for the effect of source tissue inflammation on the proliferation, differentiation, and tissue regenerative capability of IPFP MSCs and lays a foundation for future study into autologous tissue repair mechanisms and techniques.

## Conclusions

In conclusion, our results reveal that high IPFP tissue inflammation reduces the acquisition rate of MSCs and the percentage of CD34^+^ and CD146^+^ cells acquisition. However, tissue inflammation seems to not significantly influence trilineage differentiation potential and proliferative capacity of MSCs. And MSCs obtained from differing source degrees of inflammation retain stable and similar transcriptomic profile and are both safe and efficacious for tissue repair/regeneration without detectable differences. These findings provide a reference for the future clinical applications of IPFP-derived MSCs.

### Supplementary Information

**Additional file 1**. Fig S1. The macrophage marker CD68 is differentially expressed in IPFP tissue with high and low inflammation levels. Dot plot diagrams showed the number of CD68+ macrophage cells in the infrapatellar fat pad tissue with different (High and Low) inflammation levels.

**Additional file 2**. Fig S2. Gene expression patterns in ADMSCs from IPFP tissue with high and low inflammation levels. A The boxplot shows the distribution of gene expression levels for each sample, with the Y-axis for log10. The boxplot for each region corresponds to five statistics (top to down, upper limit, upper quartile, median, lower quartile and lower limit, where upper and lower limits do not consider outliers). B Volcano plots of differentially expressed genes.

**Additional file 3**. Table S1. The differentially upregulated genes (45) in MSCs of lowly inflamed tissue origin. Table S2. The differentially downregulated genes (61) in MSCs of lowly inflamed tissue origin.

## Data Availability

All data generated and/or analyzed during this study are included in this published article and its supplementary information files. The data of RNA-seq had been deposited in NCBI Sequence Read Archive (SRA) under accession No. PRJNA1018480 (https://www.ncbi.nlm.nih.gov/sra/?term=PRJNA1018480), and in China National GeneBank (CNGB) with the accession No. CNP0004293 (https://db.cngb.org/search/project/CNP0004293/). Additional experimental details and more detailed data used or analyzed during the current study are available from the corresponding author upon reasonable request.

## Abbreviation

MSCs: Mesenchymal stem cells
hPSCs: Human perivascular stem cells
hMSCs: Human mesenchymal stem cells
SVF: Stromal vascular fraction
IPFP: Human infrapatellar fat pad
IPFSCs: IPFP-derived MSCs
TGF-β1: Transforming growth factor-β1
ADMSCs: Adipose-derived mesenchymal stem cells
P/S: Penicillin/streptomycin
DMEM: Dulbecco’s modified Eagle’s medium
FBS: Fetal bovine serum
HE: Hematoxylin and eosin
CEBPA: CCAAT/enhancer binding protein, alpha
FABP4: Fatty acid binding protein 4
PPAGR2: Peroxisome proliferator-activated receptor gamma 2
ALPL: Alkaline phosphatase from liver/bone/kidney
Ocn: Osteocalcin
Runx2: Runt-related transcription factor 2
ACAN: Aggrecan
Sox 9: Sex-determining region Y-box 9
GST: Gamma glutamyl transpeptidase
ALB: Albumin
PSCs: Perivascular stem cells
FACS: Fluorescence activated cell sorting
TNF-α: Tumor necrosis factor alpha

## References

[1] Chen X, Wang S, Cao W (2018). Mesenchymal stem cell-mediated immunomodulation in cell therapy of neurodegenerative diseases. Cell Immunol.

[2] Sun Y, Chen S, Pei M (2018). Comparative advantages of infrapatellar fat pad: an emerging stem cell source for regenerative medicine. Rheumatol (Oxf).

[3] Barry F, Murphy M (2013). Mesenchymal stem cells in joint disease and repair. Nat Rev Rheumatol.

[4] Mochizuki T, Muneta T, Sakaguchi Y, Nimura A, Yokoyama A, Koga H, Sekiya I (2006). Higher chondrogenic potential of fibrous synovium- and adipose synovium-derived cells compared with subcutaneous fat-derived cells: distinguishing properties of mesenchymal stem cells in humans. Arthritis Rheum.

[5] Park JS, Yang HN, Woo DG, Jeon SY, Park KH (2011). The promotion of chondrogenesis, osteogenesis, and adipogenesis of human mesenchymal stem cells by multiple growth factors incorporated into nanosphere-coated microspheres. Biomaterials.

[6] Daneshmandi S, Karimi MH, Pourfathollah AA (2017). TGF-β1 transduced mesenchymal stem cells have profound modulatory effects on DCs and T cells. Iran J Immunol.

[7] Diehl R, Ferrara F, Muller C, Dreyer AY, McLeod DD, Fricke S, Boltze J (2017). Immunosuppression for in vivo research: state-of-the-art protocols and experimental approaches. Cell Mol Immunol.

[8] Billon N, Iannarelli P, Monteiro MC, Glavieux-Pardanaud C, Richardson WD, Kessaris N, Dani C, Dupin E (2007). The generation of adipocytes by the neural crest. Development.

[9] Vahedi P, Moghaddamshahabi R, Webster TJ, Calikoglu Koyuncu AC, Ahmadian E, Khan WS, Jimale Mohamed A, Eftekhari A (2021). The use of infrapatellar fat pad-derived mesenchymal stem cells in articular cartilage regeneration: a review. Int J Mol Sci.

[10] Di Martino A, Kon E, Perdisa F, Sessa A, Filardo G, Neri MP, Bragonzoni L, Marcacci M (2015). Surgical treatment of early knee osteoarthritis with a cell-free osteochondral scaffold: results at 24 months of follow-up. Injury.

[11] Siciliano C, Bordin A, Ibrahim M, Chimenti I, Cassiano F, Gatto I, Mangino G, Coccia A, Miglietta S, Bastianelli D (2016). The adipose tissue of origin influences the biological potential of human adipose stromal cells isolated from mediastinal and subcutaneous fat depots. Stem Cell Res.

[12] Han Y, Li H, Zhou R, Wu J, Liu Z, Wang H, Shao J, Chen Y, Zhu J, Fu Q (2021). Comparison between intra-articular injection of infrapatellar fat pad (IPFP) cell concentrates and IPFP-mesenchymal stem cells (MSCs) for cartilage defect repair of the knee joint in rabbits. Stem Cells Int.

[13] Liu Y, Holmes C (2021). Tissue regeneration capacity of extracellular vesicles isolated from bone marrow-derived and adipose-derived mesenchymal stromal/stem cells. Front Cell Dev Biol.

[14] Zhang J, Liu Y, Chen Y, Yuan L, Liu H, Wang J, Liu Q, Zhang Y (2020). Adipose-derived stem cells: current applications and future directions in the regeneration of multiple tissues. Stem Cells Int.

[15] Wu J, Kuang L, Chen C, Yang J, Zeng WN, Li T, Chen H, Huang S, Fu Z, Li J (2019). miR-100-5p-abundant exosomes derived from infrapatellar fat pad MSCs protect articular cartilage and ameliorate gait abnormalities via inhibition of mTOR in osteoarthritis. Biomaterials.

[16] Greif DN, Kouroupis D, Murdock CJ, Griswold AJ, Kaplan LD, Best TM, Correa D (2020). Infrapatellar fat pad/synovium complex in early-stage knee osteoarthritis: potential new target and source of therapeutic mesenchymal stem/stromal cells. Front Bioeng Biotechnol.

[17] Bravo B, Guisasola MC, Vaquero J, Tirado I, Gortazar AR, Forriol F (2019). Gene expression, protein profiling, and chemotactic activity of infrapatellar fat pad mesenchymal stem cells in pathologies of the knee joint. J Cell Physiol.

[18] Kouroupis D, Bowles AC, Best TM, Kaplan LD, Correa D (2020). CD10/neprilysin enrichment in infrapatellar fat pad-derived mesenchymal stem cells under regulatory-compliant conditions: implications for efficient synovitis and fat pad fibrosis reversal. Am J Sports Med.

[19] Kouroupis D, Willman MA, Best TM, Kaplan LD, Correa D (2021). Infrapatellar fat pad-derived mesenchymal stem cell-based spheroids enhance their therapeutic efficacy to reverse synovitis and fat pad fibrosis. Stem Cell Res Ther.

[20] Ioan-Facsinay A, Kloppenburg M (2013). An emerging player in knee osteoarthritis: the infrapatellar fat pad. Arthritis Res Ther.

[21] Clockaerts S, Bastiaansen-Jenniskens YM, Runhaar J, Van Osch GJVM, Van Offel JF, Verhaar JAN, De Clerck LS, Somville J (2010). The infrapatellar fat pad should be considered as an active osteoarthritic joint tissue: a narrative review. Osteoarthr Cartil.

[22] Dragoo JL, Johnson C, McConnell J (2012). Evaluation and treatment of disorders of the infrapatellar fat pad. Sports Med.

[23] Gallagher J, Tierney P, Murray P, O’Brien M (2005). The infrapatellar fat pad: anatomy and clinical correlations. Knee Surg Sports Traumatol Arthrosc.

[24] Ibrahim MM (2010). Subcutaneous and visceral adipose tissue: structural and functional differences. Obes Rev.

[25] Bartold PM, McCulloch CA, Narayanan AS, Pitaru S (2000). Tissue engineering: a new paradigm for periodontal regeneration based on molecular and cell biology. Periodontol.

[26] Intini G (2010). Future approaches in periodontal regeneration: gene therapy, stem cells, and RNA interference. Dent Clin North Am.

[27] Yao S, Pan F, Prpic V, Wise GE (2008). Differentiation of stem cells in the dental follicle. J Dent Res.

[28] Wickham MQ, Erickson GR, Gimble JM, Vail TP, Guilak F (2003). Multipotent stromal cells derived from the infrapatellar fat pad of the knee. Clin Orthop Relat Res.

[29] Squillaro T, Peluso G, Galderisi U (2016). Clinical trials with mesenchymal stem cells: an update. Cell Transpl.

[30] Qiao C, Xu W, Zhu W, Hu J, Qian H, Yin Q, Jiang R, Yan Y, Mao F, Yang H (2008). Human mesenchymal stem cells isolated from the umbilical cord. Cell Biol Int.

[31] Yan Y, Xu W, Qian H, Si Y, Zhu W, Cao H, Zhou H, Mao F (2009). Mesenchymal stem cells from human umbilical cords ameliorate mouse hepatic injury in vivo. Liver Int.

[32] Xu H, Qian H, Zhu W, Zhang X, Yan Y, Mao F, Wang M, Xu H, Xu W (2012). Mesenchymal stem cells relieve fibrosis of Schistosoma japonicum-induced mouse liver injury. Exp Biol Med (Maywood).

[33] Cao H, Qian H, Xu W, Zhu W, Zhang X, Chen Y, Wang M, Yan Y, Xie Y (2010). Mesenchymal stem cells derived from human umbilical cord ameliorate ischemia/reperfusion-induced acute renal failure in rats. Biotechnol Lett.

[34] Jiang W, Tan Y, Cai M, Zhao T, Mao F, Zhang X, Xu W, Yan Z, Qian H, Yan Y (2018). Human umbilical cord MSC-derived exosomes suppress the development of CCl4-induced liver injury through antioxidant effect. Stem Cells Int.

[35] Jacobson JA, Lenchik L, Ruhoy MK, Schweitzer ME, Resnick D (1997). MR imaging of the infrapatellar fat pad of Hoffa. Radiographics.

[36] Fawzy El-Sayed KM, Elahmady M, Adawi Z, Aboushadi N, Elnaggar A, Eid M, Hamdy N, Sanaa D, Dörfer CE (2018). The periodontal stem/progenitor cell inflammatory-regenerative cross talk: a new perspective. J Periodontal Res.

[37] Pizzute T, Lynch K, Pei M (2015). Impact of tissue-specific stem cells on lineage-specific differentiation: a focus on the musculoskeletal system. Stem Cell Rev Rep.

[38] Liu D, Xu J, Liu O, Fan Z, Liu Y, Wang F, Ding G, Wei F, Zhang C, Wang S (2012). Mesenchymal stem cells derived from inflamed periodontal ligaments exhibit impaired immunomodulation. J Clin Periodontol.

[39] Yazid FB, Gnanasegaran N, Kunasekaran W, Govindasamy V, Musa S (2014). Comparison of immunodulatory properties of dental pulp stem cells derived from healthy and inflamed teeth. Clin Oral Investig.

[40] Pereira LO, Rubini MR, Silva JR, Oliveira DM, Silva IC, Pocas-Fonseca MJ, Azevedo RB (2012). Comparison of stem cell properties of cells isolated from normal and inflamed dental pulps. Int Endod J.

[41] Lopa S, Colombini A, Stanco D, de Girolamo L, Sansone V, Moretti M (2014). Donor-matched mesenchymal stem cells from knee infrapatellar and subcutaneous adipose tissue of osteoarthritic donors display differential chondrogenic and osteogenic commitment. Eur Cell Mater.

[42] Crisan M, Yap S, Casteilla L, Chen CW, Corselli M, Park TS, Andriolo G, Sun B, Zheng B, Zhang L (2008). A perivascular origin for mesenchymal stem cells in multiple human organs. Cell Stem Cell.

[43] Esteves CL, Sheldrake TA, Mesquita SP, Pesantez JJ, Menghini T, Dawson L, Peault B, Donadeu FX (2017). Isolation and characterization of equine native MSC populations. Stem Cell Res Ther.

[44] James AW, Zhang X, Crisan M, Hardy WR, Liang P, Meyers CA, Lobo S, Lagishetty V, Childers MK, Asatrian G (2017). Isolation and characterization of canine perivascular stem/stromal cells for bone tissue engineering. PLoS ONE.

[45] Cui Z, Li C, Jiang N, Zhang C, Wang Y, Gao H, Zhou Y (2018). Isolation and characterization of minipig perivascular stem cells for bone tissue engineering. Mol Med Rep.

[46] Corselli M, Chen CW, Sun B, Yap S, Rubin JP, Peault B (2012). The tunica adventitia of human arteries and veins as a source of mesenchymal stem cells. Stem Cells Dev.

[47] Lee S, Zhang X, Shen J, James AW, Chung CG, Hardy R, Li C, Girgius C, Zhang Y, Stoker D (2015). Brief report: human perivascular stem cells and Nel-Like protein-1 synergistically enhance spinal fusion in osteoporotic rats. Stem Cells.

[48] West CC, Hardy WR, Murray IR, James AW, Corselli M, Pang S, Black C, Lobo SE, Sukhija K, Liang P (2016). Prospective purification of perivascular presumptive mesenchymal stem cells from human adipose tissue: process optimization and cell population metrics across a large cohort of diverse demographics. Stem Cell Res Ther.

[49] Bourin P, Bunnell BA, Casteilla L, Dominici M, Katz AJ, March KL, Redl H, Rubin JP, Yoshimura K, Gimble JM (2013). Stromal cells from the adipose tissue-derived stromal vascular fraction and culture expanded adipose tissue-derived stromal/stem cells: a joint statement of the international federation for adipose therapeutics and science (IFATS) and the international society for cellular therapy (ISCT). Cytotherapy.

[50] Kohla S, Ibrahim FA, Mudawi D, Akiki S, Soliman D, Al-Sabbagh A, Youssef RRH, Yassin MA (2020). High-grade epstein-barr virus-negative biphenotypic lymphoma with expression of B- and T-cell markers and leukemia presentation: case report and literature review. Case Rep Oncol.

[51] Hashino S, Takahashi S, Morita R, Kanamori H, Onozawa M, Kawamura T, Kahata K, Kondo T, Tokimatsu I, Sugita T (2013). Fungemia due to Trichosporon dermatis in a patient with refractory Burkitt’s leukemia. Blood Res.

[52] Barros MH, Hauck F, Dreyer JH, Kempkes B, Niedobitek G (2013). Macrophage polarisation: an immunohistochemical approach for identifying M1 and M2 macrophages. PLoS ONE.

[53] de Almeida DC, Donizetti-Oliveira C, Barbosa-Costa P, Origassa CS, Câmara NO (2013). In search of mechanisms associated with mesenchymal stem cell-based therapies for acute kidney injury. Clin Biochem Rev.

[54] Zielniok K, Burdzinska A, Murcia Pienkowski V, Koppolu A, Rydzanicz M, Zagozdzon R, Paczek L (2021). Gene expression profile of human mesenchymal stromal cells exposed to hypoxic and pseudohypoxic preconditioning-an analysis by RNA sequencing. Int J Mol Sci.

[55] Cho KA, Woo SY, Seoh JY, Han HS, Ryu KH (2012). Mesenchymal stem cells restore CCl4-induced liver injury by an antioxidative process. Cell Biol Int.

[56] He F, Liu X, Xiong K, Chen S, Zhou L, Cui W, Pan G, Luo ZP, Pei M, Gong Y (2014). Extracellular matrix modulates the biological effects of melatonin in mesenchymal stem cells. J Endocrinol.

[57] Li C, Li G, Liu M, Zhou T, Zhou H (2016). Paracrine effect of inflammatory cytokine-activated bone marrow mesenchymal stem cells and its role in osteoblast function. J Biosci Bioeng.

[58] Alongi DJ, Yamaza T, Song Y, Fouad AF, Romberg EE, Shi S, Tuan RS, Huang GT (2010). Stem/progenitor cells from inflamed human dental pulp retain tissue regeneration potential. Regen Med.

[59] Liu N, Shi S, Deng M, Tang L, Zhang G, Liu N, Ding B, Liu W, Liu Y, Shi H (2011). High levels of beta-catenin signaling reduce osteogenic differentiation of stem cells in inflammatory microenvironments through inhibition of the noncanonical Wnt pathway. J Bone Miner Res.

[60] Schizas NP, Zafeiris C, Neri AA, Anastasopoulos PP, Papaioannou NA, Dontas IA (2021). Inhibition versus activation of canonical Wnt-signaling, to promote chondrogenic differentiation of mesenchymal stem cells. A review. Orthop Rev (Pavia).

[61] Yao X, Mao Y, Wu D, Zhu Y, Lu J, Huang Y, Guo Y, Wang Z, Zhu S, Li X (2021). Exosomal circ_0030167 derived from BM-MSCs inhibits the invasion, migration, proliferation and stemness of pancreatic cancer cells by sponging miR-338-5p and targeting the Wif1/Wnt8/beta-catenin axis. Cancer Lett.

[62] Wang Y, Li YP, Paulson C, Shao JZ, Zhang X, Wu M, Chen W (2014). Wnt and the Wnt signaling pathway in bone development and disease. Front Biosci (Landmark Ed).

[63] Hla T, Lee MJ, Ancellin N, Paik JH, Kluk MJ (2001). Lysophospholipids–receptor revelations. Science.

[64] Pober JS, Sessa WC (2007). Evolving functions of endothelial cells in inflammation. Nat Rev Immunol.

[65] Wei S, Kitaura H, Zhou P, Ross FP, Teitelbaum SL (2005). IL-1 mediates TNF-induced osteoclastogenesis. J Clin Investig.

[66] Wang L, Zhang J, Wang C, Qi Y, Du M, Liu W, Yang C, Yang P (2017). Low concentrations of TNF-alpha promote osteogenic differentiation via activation of the ephrinB2-EphB4 signalling pathway. Cell Prolif.

[67] Lipsky PE, van der Heijde DM, St Clair EW, Furst DE, Breedveld FC, Kalden JR, Smolen JS, Weisman M, Emery P, Feldmann M (2000). Infliximab and methotrexate in the treatment of rheumatoid arthritis. Anti-tumor necrosis factor trial in rheumatoid arthritis with concomitant Therapy Study Group. N Engl J Med.

[68] Gilbert LC, Rubin J, Nanes MS (2005). The p55 TNF receptor mediates TNF inhibition of osteoblast differentiation independently of apoptosis. Am J Physiol Endocrinol Metab.

[69] Collon K, Bell JA, Gallo MC, Chang SW, Bougioukli S, Sugiyama O, Tassey J, Hollis R, Heckmann N, Oakes DA (2023). Influence of donor age and comorbidities on transduced human adipose-derived stem cell in vitro osteogenic potential. Gene Ther.

[70] Winkler S, Hempel M, Bruckner S, Tautenhahn HM, Kaufmann R, Christ B (2016). Identification of pathways in liver repair potentially targeted by secretory proteins from human mesenchymal stem cells. Int J Mol Sci.

[71] Zhang Z, Lin H, Shi M, Xu R, Fu J, Lv J, Chen L, Lv S, Li Y, Yu S (2012). Human umbilical cord mesenchymal stem cells improve liver function and ascites in decompensated liver cirrhosis patients. J Gastroenterol Hepatol.

[72] Prockop DJ (2007). “Stemness” does not explain the repair of many tissues by mesenchymal stem/multipotent stromal cells (MSCs). Clin Pharmacol Ther.

[73] Sagaradze GD, Basalova NA, Efimenko AY, Tkachuk VA (2020). Mesenchymal stromal cells as critical contributors to tissue regeneration. Front Cell Dev Biol.

[74] Huri PY, Hamsici S, Ergene E, Huri G, Doral MN (2018). Infrapatellar fat pad-derived stem cell-based regenerative strategies in orthopedic surgery. Knee Surg Relat Res.

