# Supplementary figures and images for: The influence of inflammation on the characteristics of adipose-derived mesenchymal stem cells (ADMSCs) and tissue repair capability in a hepatic injury mouse model
Source: Stem Cell Res Ther. 2023 Nov 19;14:334. doi: 10.1186/s13287-023-03532-z (PMC10659042; doi:10.1186/s13287-023-03532-z)

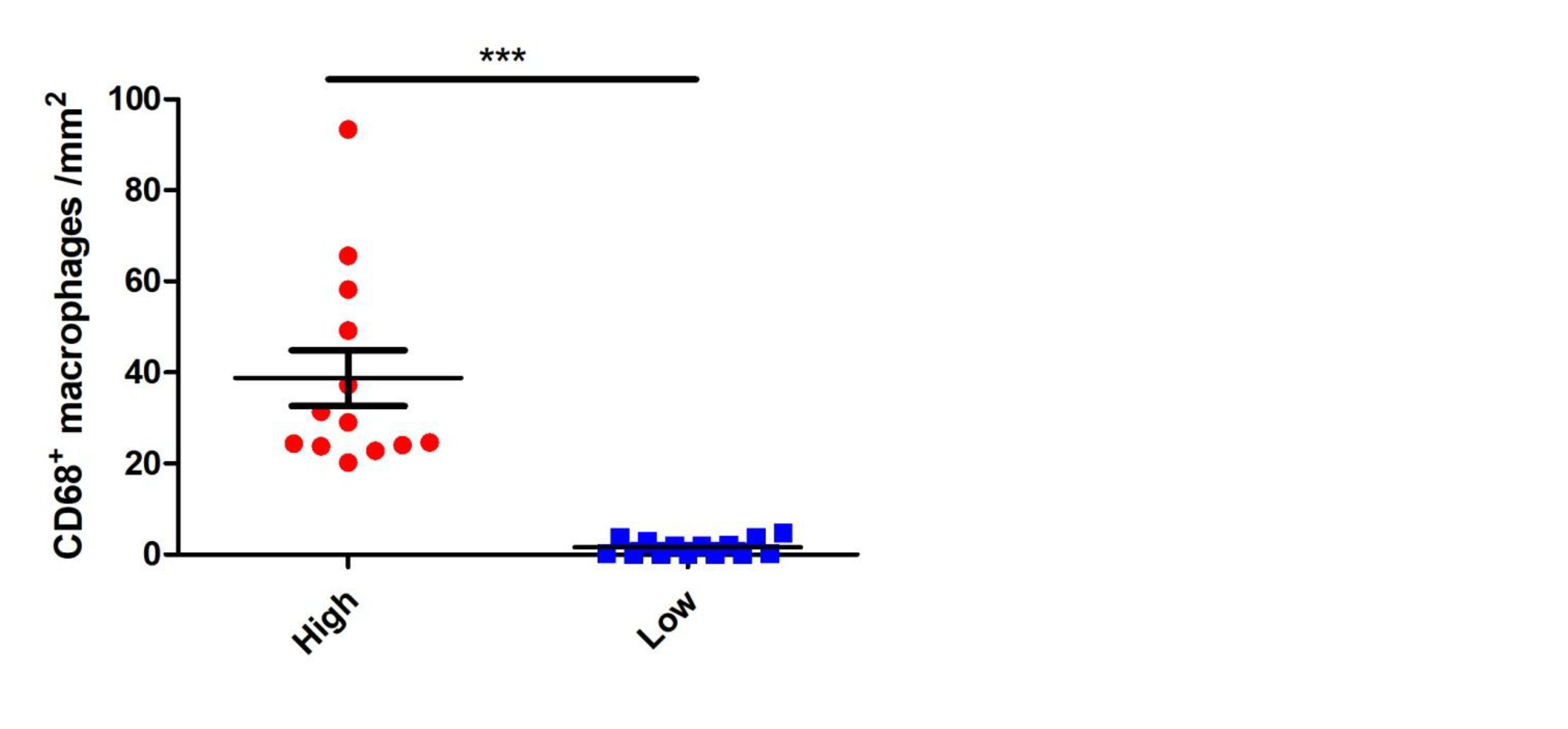

Supplement: Supplementary file 1 — Additional file 1. Fig S1. The macrophage marker CD68 is differentially expressed in IPFP tissue with high and low inflammation levels. Dot plot diagrams showed the number of CD68+ macrophage cells in the infrapatellar fat pad tissue with different (High and Low) inflammation levels. [file 13287_2023_3532_MOESM1_ESM.tif]

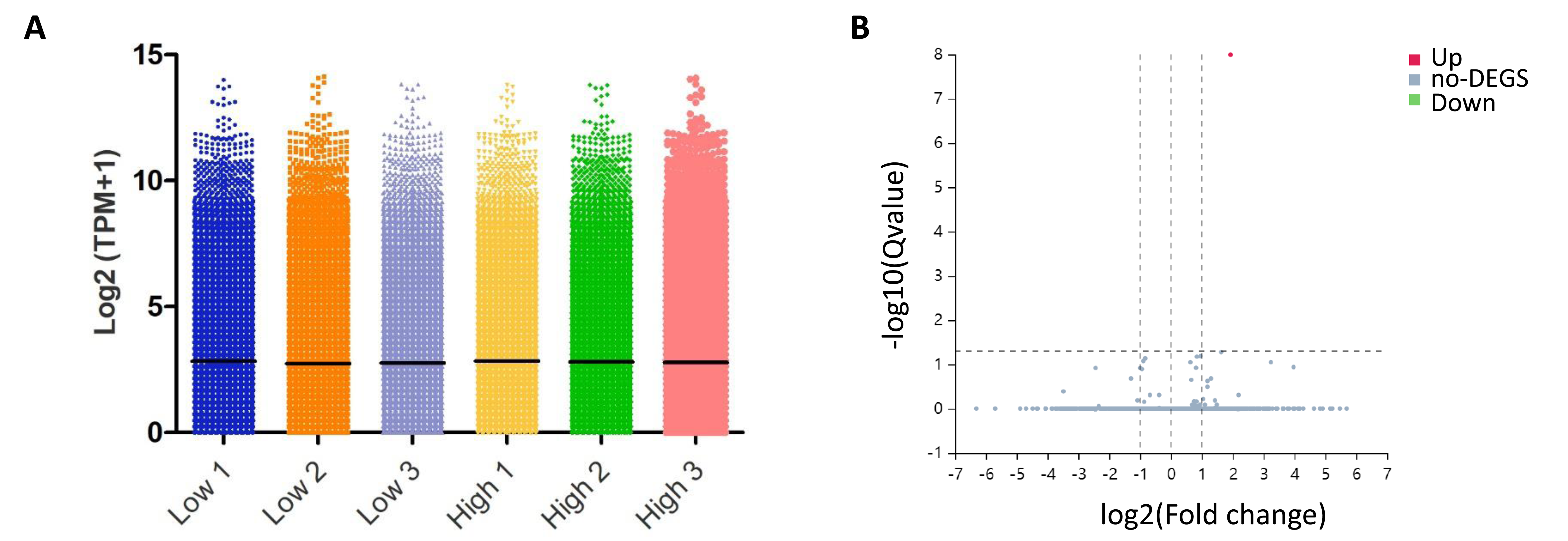

Supplement: Supplementary file 2 — Additional file 2. Fig S2. Gene expression patterns in ADMSCs from IPFP tissue with high and low inflammation levels. A The boxplot shows the distribution of gene expression levels for each sample, with the Y-axis for log10. The boxplot for each region corresponds to five statistics (top to down, upper limit, upper quartile, median, lower quartile and lower limit, where upper and lower limits do not consider outliers). B Volcano plots of differentially expressed genes. [file 13287_2023_3532_MOESM2_ESM.tif]
