# Supplementary material for: The influence of inflammation on the characteristics of adipose-derived mesenchymal stem cells (ADMSCs) and tissue repair capability in a hepatic injury mouse model
Source: Stem Cell Res Ther. 2023 Nov 19;14:334. doi: 10.1186/s13287-023-03532-z (PMC10659042; doi:10.1186/s13287-023-03532-z)
Supplement: Supplementary file 3 — Additional file 3. Table S1. The differentially upregulated genes (45) in MSCs of lowly inflamed tissue origin. Table S2. The differentially downregulated genes (61) in MSCs of lowly inflamed tissue origin. [file 13287_2023_3532_MOESM3_ESM.docx]

**Supplementary Tables**

Table S1 The differentially upregulated genes (45) in MSCs of lowly inflamed tissue origin

| **Gene Symbol** | **log2**  **(High/Low)** | **Gene Symbol** | **log2**  **(High / Low)** | **Gene Symbol** | **log2**  **(High / Low)** |
| --- | --- | --- | --- | --- | --- |
| SHC3 | -1.05 | LAMC2 | -1.42 | MMP3 | -2.44 |
| MRAP2 | -1.05 | CXCL1 | -1.51 | CYP21A2 | -2.66 |
| TENM3 | -1.07 | ECHDC3 | -1.54 | CCL5 | -2.77 |
| PLCB4 | -1.07 | FGD5 | -1.56 | TRH | -2.83 |
| IL13RA2 | -1.09 | MAMDC2 | -1.57 | TRPC6 | -2.86 |
| GREM1 | -1.09 | B3GNT5 | -1.57 | GALNT17 | -3.18 |
| CFAP58 | -1.09 | CXCL6 | -1.61 | ITM2A | -3.27 |
| EDNRB | -1.16 | SCAMP5 | -1.69 | PSG4 | -3.32 |
| STXBP6 | -1.16 | MCTP1 | -1.80 | U2AF1L5 | -3.48 |
| PLP1 | -1.20 | WNK4 | -1.81 | TSTD1 | -3.49 |
| F2RL2 | -1.25 | SPP1 | -1.87 | TBC1D3I | -3.55 |
| ANGPT1 | -1.29 | TLL1 | -1.94 | EFHB | -3.58 |
| CTSC | -1.29 | BCHE | -2.21 | RARB | -4.48 |
| LRRC17 | -1.30 | EREG | -2.34 | SIX3 | -4.89 |
| ESYT3 | -1.32 | KRTAP2-3 | -2.43 | NTS | -6.31 |

Table S2 The differentially downregulated genes (61) in MSCs of lowly inflamed tissue origin

| **Gene Symbol** | **log2**  **(High / Low)** | **Gene Symbol** | **log2**  **(High / Low)** | **Gene Symbol** | **log2**  **(High / Low)** |
| --- | --- | --- | --- | --- | --- |
| UTP14C | 5.48 | S100B | 1.86 | EFHD1 | 1.36 |
| CORO7-PAM16 | 5.23 | GPAT2 | 1.81 | ST6GAL1 | 1.30 |
| RIMBP3C | 4.64 | IRX1 | 1.79 | TFAP2A | 1.28 |
| HLA-DOB' | 4.12 | DIRAS2 | 1.75 | SORCS2 | 1.26 |
| NPTX2 | 3.97 | PLAC8 | 1.73 | GRIN2A | 1.18 |
| GIPR | 3.29 | SGCG | 1.72 | FNDC1 | 1.18 |
| LHX9 | 3.24 | LY75-CD302 | 1.67 | MCOLN3 | 1.18 |
| CHRFAM7A | 3.18 | LDLRAD4 | 1.66 | CXCL16 | 1.18 |
| PITX2 | 3.10 | PCDHGC4 | 1.64 | ADRA2A | 1.16 |
| AGT | 2.80 | GRIN3A | 1.63 | EDN1 | 1.12 |
| TSPAN18 | 2.79 | VCAM1 | 1.63 | FAM160A1 | 1.10 |
| IRX6 | 2.77 | CHD5 | 1.60 | BARX1 | 1.10 |
| ITGB2 | 2.63 | SERPINA1 | 1.59 | CEBPA | 1.09 |
| CACNA2D2 | 2.44 | TYW1B | 1.52 | ST6GALNAC3 | 1.09 |
| TSPAN8 | 2.42 | CPXM2 | 1.48 | CRACR2B | 1.08 |
| SHOX | 2.22 | CERS1 | 1.43 | TNFRSF21 | 1.08 |
| GJA3 | 2.22 | PAX9 | 1.42 | CCDC8 | 1.04 |
| TRIM67 | 2.19 | IL17RB | 1.41 | NDRG4 | 1.04 |
| C1QL1 | 1.96 | GATD3A | 1.38 | PSG5 | 1.03 |
| MAGED4B | 1.93 | SLC4A11 | 1.37 | RHPN2 | 1.02 |
| CHDH | 1.02 |  |  |  |  |
